# A New High Entropy Glycerate for High Performance Oxygen Evolution Reaction

**DOI:** 10.1002/advs.202002446

**Published:** 2021-01-27

**Authors:** Thi Xuyen Nguyen, Yen‐Hsun Su, Chia‐Chun Lin, Jrjeng Ruan, Jyh‐Ming Ting

**Affiliations:** ^1^ Department of Materials Science and Engineering National Cheng Kung University 1 University Road Tainan 70101 Taiwan

**Keywords:** electrocatalyst, high entropy glycerate, oxygen evolution reaction

## Abstract

Herein, a new high entropy material is reported, i.e., a noble metal‐free high entropy glycerate (HEG), synthesized via a simple solvothermal process. The HEG consists of 5 different metals of Fe, Ni, Co, Cr, and Mn. The unique glycerate structure exhibits an excellent oxygen evolution reaction (OER) activity with a low overpotential of 229 and 278 mV at current densities of 10 and 100 mA cm^−2^, respectively, in 1 m KOH electrolyte, outperforming its subsystems of binary‐, ternary‐, and quaternary‐metal glycerates. The HEG also shows outstanding stability and durability in the alkaline electrolyte. The result demonstrates the significance of synergistic effect that gives additional freedoms to modify the electronic structure and coordination environment. Moreover, HEG@HEG electrolyzer shows a good overall water splitting performance and durability, requiring a cell voltage of 1.63 V to achieve a current density of 10 mA cm^−2^.

## Graphical abstract

Noble metal‐free high entropy glycerate (HEG) shows robust electrolytic activity and excellent electrochemical stability for oxygen evolution reaction (OER). The OER performance of the HEG is better than its subsystems of quaternary‐, ternary‐, and binary‐metal glycerates. The excellent catalytic activity is attributed to the synergistic effects; while the excellent electrochemical stability is ascribed to the high entropy configuration.

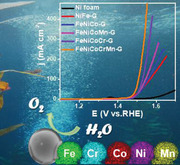

## 1 Introduction

Oxygen evolution reaction (OER) plays a key role in energy storage devices, such as rechargeable metal air battery and energy generation through electrochemical water‐splitting.^[^ ^1^, ^2^, ^3^ ^]^ However, OER suffers from its sluggish reaction kinetics. Solving the issues related to the sluggish reaction kinetics is not trivial. In one of the scenarios, i.e., electrocatalytic hydrogen production, a major issue is to reduce the overpotential to accelerate the kinetics of the reaction. Up to date, ruthenium oxide (RuO_2_) and iridium oxide (IrO_2_) are the most active OER electrocatalysts.^[^ ^4^, ^5^ ^]^ Unfortunately, the high price and scarcity make their large‐scale applications undesirable. The search for earth‐abundant metal‐based materials as alternative OER electrocatalysts has thus been called for. Currently, studies are being made to improve the performance of transition‐metals based OER electrocatalysts. Among them, the first‐row transition metal hydroxides/(oxy)hydroxides have been recognized as the most efficient and promising materials for enhancing OER activity in alkaline media.^[^ ^6^, ^7^ ^]^ Hydroxides or (oxy)hydroxides of double metals, such as NiFe and CoFe, have been shown to be highly active OER electrocatalysts that exhibit low overpotentials.^[^ ^6^, ^8^, ^9^, ^10^ ^]^ Moreover, the introduction of a third metal, such as V, Cr, Mo, further improves the OER activity.^[^ ^11^, ^12^, ^13^, ^14^ ^]^ This can be understood since the catalytic activity or performance strongly depends on the chemical composition and surface electronic structure. Increasing the complexity of the constituents to enhance the catalytic performance certainly represents a new territory and is thus under intensive explorations. A very recent model example is the ever‐growing high entropy materials (HEMs) that consist of five or more elements.

HEMs have attracted significant attentions since the 2004 pioneering reports on high entropy alloys (HEAs) by Yeh et al. and Cantor et al.^[^ ^15^, ^16^ ^]^ With the large composition space, HEAs exhibit several improved or unexpected properties. HEMs other than HEAs, including high entropy metallic glasses,^[^ ^17^, ^18^ ^]^ high entropy oxides (HEOs),^[^ ^19^, ^20^, ^21^ ^]^ carbides,^[^ ^22^, ^23^ ^]^ borides,^[^ ^24^, ^25^ ^]^ nitrides,^[^ ^26^ ^]^ sulfides,^[^ ^27^ ^]^ and silicides,^[^ ^28^ ^]^ have also appeared. Their excellent properties are desired for many applications.^[^ ^26^, ^29^, ^30^ ^]^ For example, unexpected physical and mechanical properties, such as high hardness, wear resistance, and great thermal and structural stability, have been found in HEAs;^[^ ^31^, ^32^ ^]^ while HEOs have been shown to have superionic conductivity,^[^ ^30^ ^]^ narrow bandgap,^[^ ^33^ ^]^ colossal dielectric constant,^[^ ^34^ ^]^ long‐range antiferromagnetic order,^[^ ^35^ ^]^ and superior lithiation‐delithiation properties.^[^ ^36^ ^]^ Of interest here is the catalytic performance of HEMs.

HEMs have been investigated for use as catalysts in various catalytic reactions, including oxygen/hydrogen evolution, oxygen reduction, ammonia decomposition, and CO, methanol, and ammonia oxidation.^[^ ^37^ ^]^ For OER, Jin et al. reported nanoporous AlNiCoIrMo HEA electrocatalyst in acidic electrolyte.^[^ ^38^ ^]^ With ≈20% Ir concentration, nanoporous AlNiCoIrMo HEA exibits a high activity with an overpotential of 233 mV at a current density of 10 mA cm^−2^ and enhanced electrochemical cycling stability. High entropy (Co, Cu, Fe, Mn, Ni)_3_O_4_ spinel oxide nanoparticles synthesized via a low temperature solvothermal process followed by annealing were mixed with multi‐walled carbon nanotubes for use as OER catalysts.^[^ ^39^ ^]^ An overpotential of 350 mV at 10 mA cm^−2^ was reported, showing the (Co, Cu, Fe, Mn, Ni)_3_O_4_ to be a promising candidate for OER application. Recently, high entropy perovskite fluorite K_0.8_Na_0.2_(MgMnFeCoNi)F_3_ was shown to exhibit excellent OER performance, having an overpotential of 314 mV at a current density of 10 mA cm^−2^.^[^ ^40^ ^]^ These HEMs exhibit multiple valence states and diverse surface characteristics, thus giving unique synergistic effects for OER. The virtually endless element combinations of HEM give enormous possibilities to tailor the surface electronic structure and hence optimize the catalytic activity. Therefore, as large as the composition space is, the gateway to the exploration of HEMs has just opened. We therefore herein report a new high entropy glycerate (HEG) based on Co, Cr, Fe, Mn, and Ni, and their use as novel, high‐performance water oxidation electrocatalyst. Metal glycerate, a typical metal alkoxide, has been used as an intermediate template to synthesize materials with different morphologies for various applications.^[^ ^41^, ^42^, ^43^, ^44^ ^]^ Metal glycerate has a layered structure that consists of stacked metal‐oxygen sheets separated by glycerate anions.^[^ ^45^ ^]^ The layered structure is similar to anion‐intercalated hydroxides, which provide desired interlayer spacing for the accommodation of reactants.^[^ ^46^ ^]^ The open structure allows rapid transport of the reactants to the material and also gives additional catalytic active sites. Furthermore, due to the similarity to the hydroxide, the formation of favored OOH functional group is expected during the OER reaction. Recently, the use of metal alkoxides in OER has been reported.^[^ ^46^, ^47^, ^48^ ^]^ For instance, binary‐metallic FeNi‐glycerate hierarchical microsphere OER electrocatalyst exhibiting an overpotential of 320 mV at 10 mA cm^−2^ and 12 h long‐term stability in a 1 m KOH aqueous solution has been reported.^[^ ^46^ ^]^ Here, there are five metals of Co, Cr, Fe, Mn, and Ni in the HEG. Among them, Co, Fe, Ni, and Mn‐based materials are known to be very active OER electrocatalysts.^[^ ^13^, ^49^, ^50^, ^51^ ^]^ Meanwhile, Cr has also been found to improve the electrocatalytic activity when incorporated into NiFe‐based catalysts.^[^ ^11^, ^12^ ^]^ The multiple active constituents in the resulting HEG, coupled with the high‐entropy stabilization effect, shows an excellent electrochemical OER performance.

## 2 Results and Discussion

**Figure 1:**
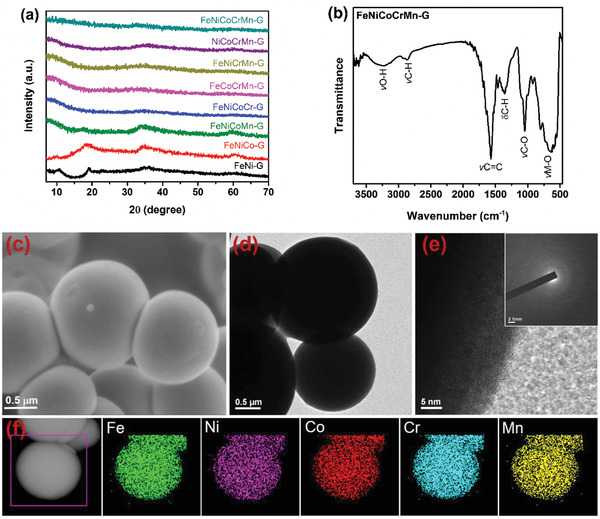
a) XRD spectra of multi‐metal glycerates, b) FTIR spectrum of FeNiCoCrMn‐G, c) SEM, and d) TEM images showing uniformsized FeNiCoCrMn‐G microspheres, e) HRTEM and SAED analyses, and f) STEM‐EDS mappings of FeNiCoCrMn‐G.

The crystalline structures of the obtained samples were first examined. **Figure** **1**a shows the X‐ray diffraction (XRD) profiles of multi‐metal glycerate samples. The binary FeNi‐G shows diffraction peaks at 10.8°, 19.2°, 35.3°, and 60°, indicating the formation of metal glycerate that consists of stacked metal‐oxygen sheets separated by bonded glycerate ions.^[^ ^45^, ^46^, ^52^ ^]^ By adding more elements, the diffraction peaks become broadened, suggesting reduced crystallinity. The crystallinity of the quaternary‐metal glycerates and HEG become almost amorphous. Fourier transform infrared spectroscopy (FTIR) analysis was conducted to further investigate the molecular structure of the samples. As shown in Figure 1b, and Figure S1 in the Supporting Information, all the samples exhibit identical spectra due to their inherent glycerol moiety. The broad IR absorption band centering at 3400 cm^−1^ is attributed to the stretching vibrations of hydrogen bonded O—H groups; while the absorption bands between 2850–2950 cm^−1^ are assigned to the C—H stretching vibrations.^[^ ^45^ ^]^ The high‐intensity band at 1580 cm^−1^ corresponds to the C=C stretching vibration.^[^ ^44^ ^]^ A peak at 1640 cm^−1^, belonging to the C=O vibration, is also seen. The IR absorption band at 1358 cm^−1^ is indexed to the C‐H bending vibration; while the band at 1100 cm^−1^ is attributed to the C‐O stretching vibration.^[^ ^53^ ^]^ The peak at about 800 cm^−1^ is assigned to the out of plane C—H bending vibrations.^[^ ^53^ ^]^ Moreover, another prominent IR band centering at 630 cm^−1^ is associated with metal‐oxygen (M—O, M presents Fe, Ni, Co, Cr, and Mn) stretching vibrations.^[^ ^45^ ^]^ FTIR and XRD results confirm the formation of multimetal glycerates. Scanning electron microscopy (SEM) and transmission electron microscopy (TEM) images show that the FeNiCoCrMn‐G HEG are microspheres, as shown in Figure  1c, and d, respectively. High resolution TEM (HRTEM) and selected area electron diffraction (SAED) (Figure 1e) analyses indicate the formation of amorphous structure, which is consistent with the XRD result. Dark field scanning transmission electron microscopy (STEM) coupled with energy‐dispersive X‐ray spectroscopy (STEM‐EDS) mappings of the FeNiCoCrMn‐G show that all the five metal elements distribute homogenously throughout the microsphere (Figure 1f). The chemical compositions of the samples were evaluated using inductively coupled plasma‐mass spectrometry (ICP‐MS), as shown in Table S1 (Supporting Information). The analysis shows that the metal concentrations are nearly equal to that of the respective precursor concentrations.

**Figure 2:**
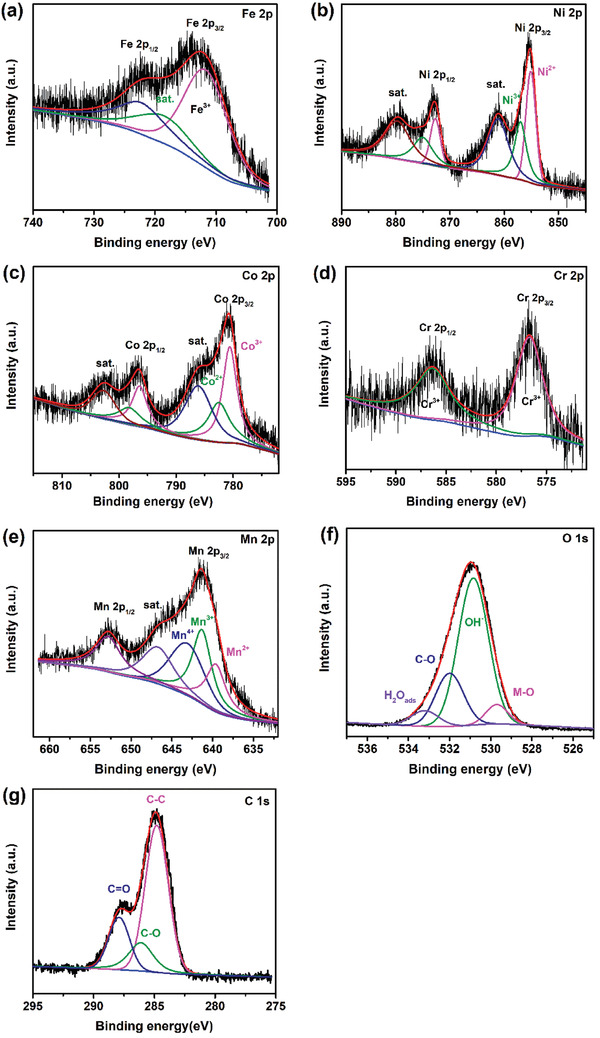
XPS detailed spectra of a) Fe 2p, b) Ni 2p, c) Co 2p, d) Cr 2p, e) Mn 2p, f) O 1s, and g) C 1s of the HEG.

To examine the surface chemistry of the obtained samples, X‐ray photoelectron spectroscopy (XPS) analysis was performed, as shown in **Figure** **2** for the FeNiCoCrMn‐G HEG. The Fe 2p spectrum shows broadened Fe 2p_3/2_ and Fe 2p_1/2_ peaks centering at 712.4 and 723.6 eV, respectively. Both belong to trivalent iron Fe^3+^.^[^ ^54^ ^]^ A satellite peak of Fe^3+^ is located at 718.4 eV. In the Ni 2p spectrum, the Ni 2p_3/2_ (Ni^2+^) and Ni 2p_1/2_ (Ni^2+^) peaks are located at 855.2 and 872.9 eV, respectively; while the Ni 2p_3/2_ (Ni^3+^) and Ni 2p_1/2_ (Ni^3+^) peaks are at 857.2 and 875.3 eV, respectively.^[^ ^55^ ^]^ Two prominent shakeup satellite peaks at 861.4 and 880.0 eV were found. Two different valence states were also found in the Co 2p spectrum. The peaks at 780.6 (Co 2p_3/2_) and 796.4 eV (Co 2p_1/2_) correspond to Co^3+^, and the peaks at 782.3 (Co 2p_3/2_) and 798.2 eV (Co 2p_1/2_) belong to Co^2+^.^[^ ^14^ ^]^ Two prominent shakeup satellite peaks are seen at 786.1 and 802.6 eV. The Cr 2p spectrum shows two peaks at 576.5 and 586.3 eV, which belong to the Cr 2p_1/2_ and Cr 2p_3/2_, respectively, indicating the existence of Cr^3+^.^[^ ^12^ ^]^ In the Mn 2p spectrum, two peaks located at 641.3 and 653.2 eV belong to the Mn 2p_1/2_ and Mn 2p_3/2_, respectively. The Mn 2p_3/2_ peak was well deconvoluted into three peaks, which are Mn^2+^ at 640 eV, Mn^3+^ at 641.35 eV, and Mn^4+^ at 643 eV.^[^ ^56^ ^]^ The peak at 647 eV is a satellite peak. The O 1s spectrum were deconvoluted into four components at 529.6, 531.3, 532, and 533.2 eV, which are assigned to the lattice oxygen O—M, O—H, C—O bonding, and physically absorbed water, respectively.^[^ ^57^, ^58^ ^]^ For the C 1s spectrum, the peaks at 284.8, 286.2, and 287.6 eV are attributed to the C—C, C—O, and C=O bonding, respectively.^[^ ^46^, ^59^ ^]^

**Figure 3:**
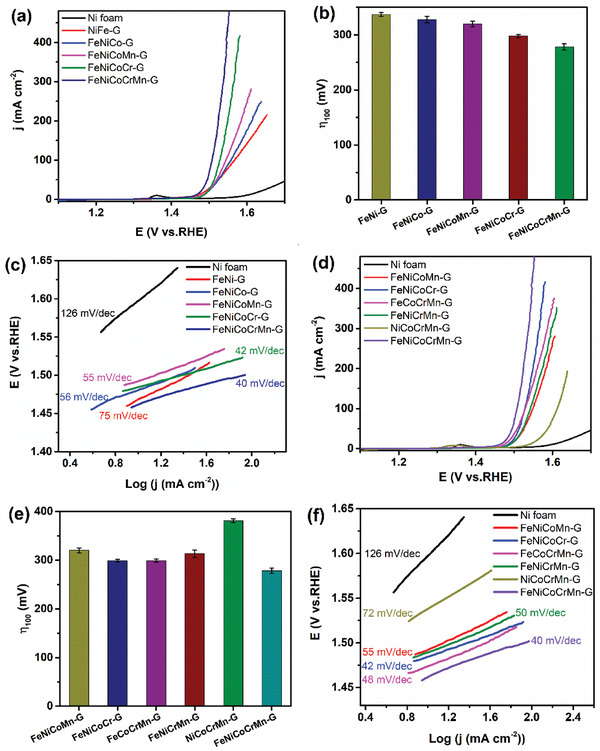
a) LSV curves, b) overpotentials at 100 mA cm^−2^, and c) the corresponding Tafel plots of Group I glycerates. d) LSV curves, e) overpotentials at 100 mA cm^−2^, and f) the corresponding Tafel plots of Group II glycerates. Error bars represent standard deviations based on triplicate measurements.

The electrocatalytic OER activity of the obtained glycerates were evaluated using a standard three‐electrode system in a 1 m KOH electrolyte. The polarization linear sweep voltammetry (LSV) curves of unary‐metal glycerates are shown in Figure S2 (Supporting Information). The Fe‐G exhibits the best activity, followed by the Co‐G and then Ni‐G. To demonstrate the OER performance can be enhanced with the addition of a different metal, Group I multi‐metal glycerates, consisting of binary‐metal FeNi‐G, ternary FeNiCo‐G, quaternary‐metal FeNiCoCr‐G and FeNiCoMn‐G, and quinary‐metal FeNiCoCrMn‐G, are first presented. The LSV curves of these multi‐metal glycerates are shown in **Figure** **3**a. To achieve a benchmark current density of 10 mA cm^−2^, the HEG electrode requires only a potential of 1.459 V versus the reversible hydrogen electrode (RHE), corresponding an excellent overpotential of 229 mV (η_10_ = 229 mV). The OER catalytic activity of the HEG electrode outperforms all the rest of the glycerate electrodes. This performance is also better than that of many reported transition metal‐based electrocatalysts (Table S2, Supporting Information). Detailed comparison of the overpotentials of the Group I glycerates at 100 mA cm^−2^ is shown in Figure 3b and **Table** **1**. It is seen that the HEG has the lowest *η*_100_ of 278 mV. The electrocatalytic activity of these glycerates follows this order: FeNiCoCrMn‐G > FeNiCoCr‐G > FeNiCoMn‐G > FeNiCo‐G > FeNi‐G. The catalytic kinetics of these glycerate electrocatalysts were also evaluated using the Tafel slopes derived from the LSV curves, as shown in Figure 3c and Table 1. The HEG gives the lowest Tafel slope of 40 mV dec^−1^, followed by the FeNiCoCr‐G (42 mV dec^−1^), and then the FeNiCoMn‐G (55 mV dec^−1^), and FeNiCo‐G (56 mV dec^−1^). The FeNi‐G exhibits a higher slope of 75 mV dec^−1^. In other words, in terms of the kinetics, the OER performances also follow the same order as the above. The addition of Fe to Ni‐G to form FeNi‐G leads to electronic structure modification, thus facilitating the OER reaction.^[^ ^46^, ^60^, ^61^, ^62^ ^]^ Further addition of metal continues to modify the electronic structure. The surface electronic structures of Fe and Ni in the Group I glycerates are shown in Figure S3 (Supporting Information). Binary FeNi‐G shows the co‐existence of Fe^2+^ and Fe^3+^, while only Fe^3+^ are seen in the ternary, quaternary, and quinary samples. The Fe 2p peak exhibits significant positive shifts with increasing number of metals. In the meantime, the Ni 2p peak also slightly shifts toward the high binding energy side and the concentration of Ni^3+^ increases with the addition of metal. The modification of the electronic structure leads to enhanced electrocatalytic activity. It is also noted that the addition of Cr enhances the activity more than the addition of Mn. The ionic radius of Cr is the largest among all the metal ions. The presence of Cr then causes strain that is favorable for OER reaction,^[^ ^6^ ^]^ due to weakened chemisorption at the active sites.^[^ ^63^ ^]^ With the addition of both Cr and Mn, the FeNiCoCrMn‐G exhibits the best OER activity. It was observed that accompanying the metal addition is the presence of metal ions with higher oxidation states, indicating the variation of local coordination environment that influences OER performance.^[^ ^6^ ^]^

**Table 1:** Comparison of catalytic performances of the obtained glycerates. The standard deviations based on triplicate measurements

Electrocatalyst	*η*_100_ [mV]	Tafel slope [mV dec^−1^]	*C*_dl_ [mF cm^−2^]
FeNi‐G	337 ± 4	75 ± 3	1.42
FeNiCo‐G	328 ± 6	56 ± 4	1.43
FeNiCoMn‐G	320 ± 5	55 ± 2	1.35
FeNiCoCr‐G	298 ± 3	42 ± 2	1.15
FeCoCrMn‐G	299 ± 3	48 ± 1	1.55
FeNiCrMn‐G	313 ± 8	50 ± 2	0.95
NiCoCrMn‐G	381 ± 4	72 ± 3	0.93
FeNiCoCrMn‐G	278 ± 5	40 ± 2	1.63

**Figure 4:**
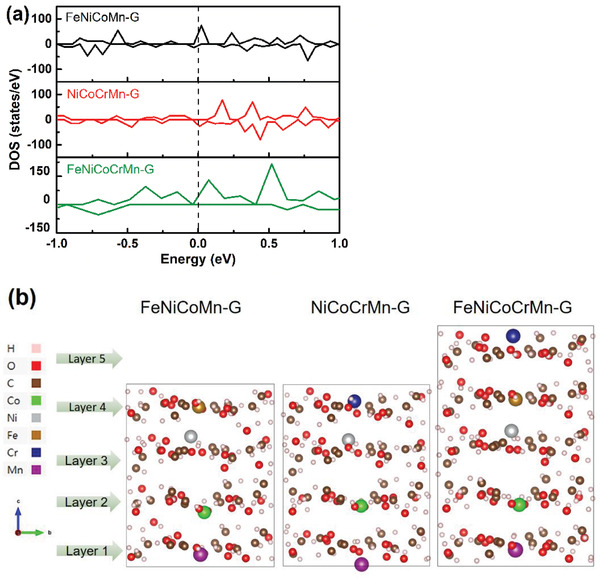
a) Density of states and b) relaxed structures of FeNiCoMn‐G, NiCoCrMn‐G, and FeNiCoCrMn‐G.

**Figure 5:**
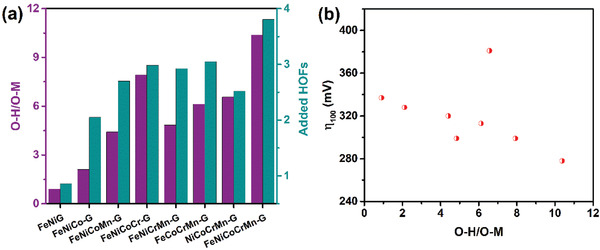
a) O—H/O—M versus added HOFs. b) *η*_100_ as function of O—H/O—M ratio.

The outstanding performance of HEG demonstrates the synergistic effects of the high entropy configuration. This can also be realized by examining the removal of each metal from the HEG. This is demonstrated by considering Group II glycerates consisting of the FeNiCoCrMn‐G HEG, and 5 quaternary‐metal glycerates of FeCoCrMn‐G, FeNiCoCr‐G, FeNiCrMn‐G, FeNiCoMn‐G, and NiCoCrMn‐G. With the removal of any element, the performance is reduced, as shown in Figure 3d,e, and f. The overpotentials and Tafel slopes are listed in Table 1. The performance in terms of the *η*_100_ shows the following order: FeNiCoCrMn‐G (278 eV) > FeNiCoCr‐G (298 eV) > FeCoCrMn‐G (299 eV) > FeNiCrMn‐G (313 eV) > FeNiCoMn‐G (337 eV) > NiCoCrMn‐G (381 eV). In terms of the Tafel slope, it is FeNiCoCrMn‐G (40 mV dec^−1^) > FeNiCoCr‐G (42 mV dec^−1^) > FeCoCrMn‐G (48 mV dec^−1^) > FeNiCrMn‐G (50 mV dec^−1^) > FeNiCoMn‐G (55 mV dec^−1^) > NiCoCrMn‐G (72 mV dec^−1^). Among the five quaternary‐metal glycerates, removing Fe from the FeNiCoCrMn‐G leads to the worst performance. It indicates that the Fe plays the key role in the OER activity of the multi‐metal glycerates. The removal of Cr leads to the second worst performance, highlighting the critical role of the aforementioned strain effect, in addition to modifying the electronic structure. To show the key role of Fe, the density of states of FeNiCoMn‐G, NiCoCrMn‐G, and FeNiCoCrMn‐G were determined. **Figure** **4**a shows that the FeNiCoCrMn‐G has more electronic states near and below the Fermi level, and is followed by FeNiCoMn‐G and then NiCoCrMn‐G. This indicates that there are more states in the valence band, giving more states of holes for the oxidation reaction. The critical role of Fe is thus confirmed. To show the addition of Cr causes additional strain, the relaxed structures of FeNiCoMn‐G, NiCoCrMn‐G, and FeNiCoCrMn‐G were examined. Different metal cations in the glycerates induce various strains in the layer structures, as shown in Figure 4a. Different metal cations in the glycerates induce different steric strains to affect the shape and conformation of ions and molecules in lattice. The strains were calculated using $\frac{\sum |X_{f}-X_{i}|}{N}$, where *X*_i and_ *X*_f_ represent the positions along the c direction before and after the relaxation, respectively, and *N* is the number of atoms in the layer. The obatined strains are shown in Table S3 (Supporting Information). The total average strains are 0.536, 0.522, and 0.516 Å per atom for NiCoCrMn‐G, FeNiCoCrMn‐G, and FeNiCoMn‐G, respectively. With the presence of Cr, additional strains are seen. To further examine the glycerates, the oxidation states of the metals are discussed. As mentioned above, all the metals in the obtained multimetal glycerates exhibit valence states of 2^+^ and 3^+^; while an additional 4^+^ was found for the Mn. The higher oxidation‐state fraction (HOF), i.e., (M^3+^+M^4+^)/(M^2+^+M^3+^+M^4+^), of each metal M was then determined from the XPS analysis. The added HOFs for all the metals in each sample are shown in **Figure** **5**a. The HEG has the highest added HOF, follow by the quaternary‐, the ternary‐, and the binary‐metal glycerates. In a glycerate, the number of coordination bonding between the metal and ligand depends on the metal oxidation state, as shown in Figure S4 (Supporting Information). In other words, for metals having valence states of 3^+^ and 4^+^, there are 3 and 4 attached ligands, respectively. This is supported by the finding that the XPS O—H/O—M ratio increases with the HOF, as also shown in Figure 5a. The variations lead to different coordination environments such that the electrocatalyst activity is increased with the O—H, as shown in Figure 5b. The only exception is the glycerate without Fe, i.e., NiCoCrMn‐G. Due to the critical role of Fe, this sample has a high value of *η*_100_ in spite of its high O—H/O—M ratio. The resulting coordination bonding is expected to give more freedoms to the glycerate anions to flip the coordination mode, thus facilitating the OER process.^[^ ^46^, ^64^ ^]^ Moreover, with a higher oxidation state or more coordination bonding, crystalline distortion (expanding) and even amorphortization can be expected. Therefore, as shown in Figure 1a, the addition of Co into FeNi‐G reduces the peak intensity and the addition of Mn into FeNiCo‐G continues to reduce the intensity. This indicates the expanding of the interlayers in the layered structure. The presence of Cr or Cr^3+^ nearly amorphortizes the glycerates. Low crystallinity or amorphous structure is known to provide more active sites and accelerate the charge transfers between the intermediates and the active sites.^[^ ^65^, ^66^, ^67^ ^]^ An amorphous structure provides more active sites, such as bonds with random orientations and unsaturated electronic configurations, favoring the adsorption of reaction species.^[^ ^66^, ^67^ ^]^ Due to the flexibility of the local structure in an amorphous structure, the charge transfer between the intermediates and the active sites can also be accelerated, which also contributes to the enhanced OER performance.^[^ ^65^ ^]^ To show this, we have annealed the amorphous FeNiCoCrMn‐G sample at different temperatures of 400 and 850 °C for 2 h in air, and then examined their OER performances. The obtained XRD patterns and LSV polarization curves are shown in Figure S5 (Supporting Information). The crystallinity of the amorphous FeNiCoCrMn‐G improves with the annealing temperature. Both the 400 and 850 °C annealed samples exhibit a single‐phase spinel oxide structure with the latter showing better crystallinity (Figure S5a, Supporting Information). Therefore, as shown in Figure S5b (Supporting Information), the OER activity reduces with the crystallinity.

**Figure 6:**
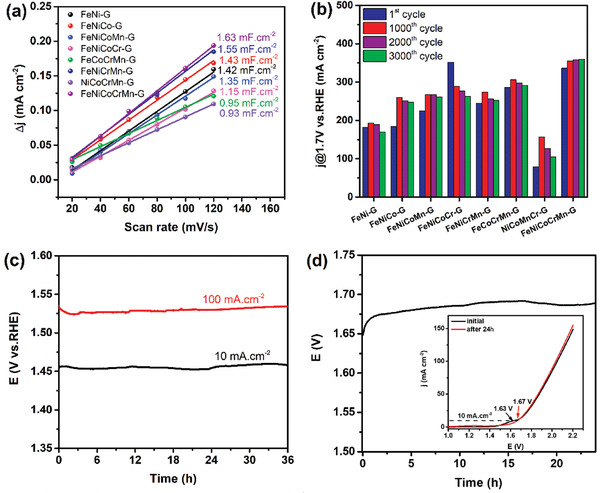
a) Double layer capacitance and b) the electrochemical stability of all the obtained multiple‐metal glycerates. The current densities at *E* = 1.7 V were recorded after the first, 1000th, 2000th, and 3000th cycles. c) Durability of the HEG at 10 and 100 mA cm^−2^ for 36 h. d) Overall water splitting activity of FeNiCoCrMn‐G@FeNiCoCrMn‐G electrolyzer.

The electrochemical active surface areas (ECSAs) of all the metal glycerates were estimated by examining the double layer capacitance (*C*_dl_). *C*_dl_ was determined by measuring the charging current density in a non‐Faradaic potential window of 0.95–1.05 V versus RHE. **Figure** **6**a shows the plots of Δ*j* = (ja–jc)/2 at *E* = 1.0 V versus RHE against the scan rates for the multiple‐metal glycerates. All the glycerates show comparable *C*_dl_ values, thus similar ECSAs, indicating their minor contribution to the OER performance. With the similar *C*_dl_, the HEG outperforms all the other samples. This again depicts that the desired high intrinsic catalytic activity and kinetics of the high entropy configuration. Electrochemical impedance spectroscopy (EIS) analysis was also performed on all glycerates. The Nyquist plots are shown in Figure S6a (Supporting Information). The charge transfer resistance (*R*_ct_) appears to decrease generally with the addition of metal. The aforementioned electronic structure modification and the variation of coordination bonging may play a role here such that the *R*_ct_ decreases with the M—OH bonding, as in Figure S6b (Supporting Information). The exact mechanism is unknown; however, the *R*_ct_ of the FeNiCoCrMn‐G HEG is the smallest, further confirming that the high entropy material gives enhanced reaction kinetics.

The long‐term electrochemical stability of all the glycerate electrodes was evaluated through continuous cyclic voltammetry (CV) cycling at a scan rate of 100 mV s^−1^ for 3000 cycles. OER activities were recorded after selected numbers of cycles. Figure 6b shows the OER activities of all the multimetal glycerate electrodes obtained after the 1st, 1000th, 2000th, and 3000th cycles. The LSV curves after selected cycles are shown in Figure S7 (Supporting Information). After 1000 cycles, all the samples show increased activities, except the FeNiCoCr‐G. After 2000 and 3000 cycles, the binary‐, ternary‐, and quaternary‐metal glycerates show decreased activities. However, the FeNiCoCrMn‐G HEG exhibits increased performance even after 3000 cycles. This verifies the excellent electrochemical stability of the HEG electrode. The electrochemical stability of the FeNiCoCrMn‐G HEG is attributed to the synergistic effect of high entropy configuration. During the cyclic tests, we observed progressive anodic redox peak shift and increased peak intensity with the cycle number until reaching a steady state, except that the Fe‐free NiCoCrMn‐G has a slight cathodic shift, as shown in Figure S8 (Supporting Information). The redox peaks correspond to the transformation of Ni(OH)_2_ to NiOOH. The increased activities after the cycling and the continuously positive shift of the redox peaks suggest the modification of chemical state and structure. The HEG electrocatalyst was further evaluated for its durability. Figure 6c shows the 36 h durability test result obtained at *j* = 10 and 100 mA cm^−2^. The sample shows no appreciable change, indicating excellent durability after 36 h. Such remarkable durability indicates that the HEG electrocatalyst is very stable during the OER. All the glycerates were also evaluated for the electrocatalytic HER activity in 1 m KOH electrolyte. The LSV curves and Tafel plots are shown in Figure S9a,b (Supporting Information), respectively. Due to the synergistic effects of multiple elements, the HEG exhibits the best performance, giving the lowest overpotential of 210 mV at 10 mA cm^−2^ and the smallest Tafel slope of 105 mV dec^−1^. The HER performance of the rest of the samples are listed in Table S4 (Supporting Information). The overall water splitting performance was further evaluated using a FeNiCoCrMn‐G@FeNiCoCrMn‐G electrolyzer. As shown in Figure 6d, the electrolyzer exhibits a good performance with a cell voltage of 1.63 V at a current density of 10 mA cm^−2^. Meanwhile, the LSV curve shows no significant change after 24 h of continuous testing, indicating the long‐term durability of the dual‐function HEG catalyst. Without optimizing the high entropy configuration, the FeNiCoCrMn‐G HEG exhibits a comparable overall water splitting performance, as shown in Table S5 (Supporting Information).

**Figure 7:**
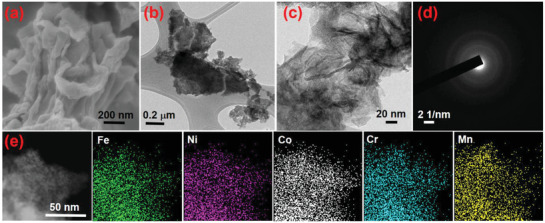
a) SEM image, b,c) TEM images, d) SAED, and e) High‐angle annular dark‐field (HAADF) and STEM‐EDS mapping images of the FeNiCoCrMn‐G after OER test.

The remarkable cyclic stability and durability root in the nature of the high entropy configuration. The HEG microspheres transformed into a porous structure consisting of thin sheets after the OER, as observed using SEM (**Figure** **7**a) and TEM (Figure 7b,c). The sheet‐like structure is amorphous, as shown in the SEAD (Figure 7d). The STEM‐EDS mappings (Figure 7e) show the uniform distributions of all the metals. The high resolution XPS spectra of the pristine and post‐OER electrocatalysts are shown in Figure S10 (Supporting Information). The Fe 2p spectrum remains unchanged with the presence of only Fe^3+^. The intensities of the Ni^3+^ and Co^3+^ peaks increase slightly. The Cr spectrum shows a positive peak shift. The Cr 2p_3/2_ peak at 577. 5 eV and Cr 2p_1/2_ at 587.2 eV belong to Cr^3+^. The Mn^2+^ was partially oxidized to higher oxidation states. The increased metal 3^+^ indicates the formation of metal oxyhydroxide.^[^ ^12^, ^46^, ^68^, ^69^ ^]^ Critically, the O 1s spectrum shows a clear transition. Before the OER, the OH^−^ peak dominates at 531.1 eV; while after the OER, the intensity of M‐O bonding at 529.6 eV increases, giving an O—M/O—H ratio of ≈1. The results supports the formation of the aforementioned metal oxyhydroxides during the OER reaction.^[^ ^68^, ^70^ ^]^ These oxyhydroxides are very likely to be the real intrinsic active species for the OER. Nevertheless, we have shown that the excellent OER activity is attributed to the nature of the glycerate and high entropy configuration, and the unique synergistic effects of the HEG. It is also believed that further modification of the FeNiCoCrMn‐G HEG by adjusting the concentration and/or addition of more metals would leads to optimized glycerates that exhibit both superior OER and overall water splitting activities.

## 3 Conclusion

In summary, amorphous, noble metal‐free HEG has been synthesized using a facial and scalable solvothermal method. We have demonstrated that the HEG, having 5 metals of Fe, Ni, Co, Cr, and Mn, is a highly active and stable electrocatalyst for OER. The HEG was found to be more active than its subsystems of quaternary‐, ternary‐, and binary‐metal glycerates. The excellent catalytic activity of the HEG is attributed to the synergistic interactions among the metal elements; while the excellent electrochemical stability is ascribed to the high entropy configuration effect. The result demonstrates a viable strategy that leads to the development of new HEG materials with excellent OER and overall water splitting performances. It also opens a new avenue to explore novel, outstanding high entropy materials for various applications.

## 4 Experimental Section

##### Chemicals

Co(NO_3_)_2_.6H_2_O (J.T. Baker, 99%), Cr(NO_3_)_2_.9H_2_O (Alfa Aesar, 98.5%), Fe(NO_3_)_3_·9H_2_O (J.T. Baker, 99%), Ni(NO_3_)_3_·6H_2_O (Alfa Aesar, 98.5%), Mn(NO_3_)_2_.6H_2_O (Alfa Aesar, 98.5%), glycerol (Macron, 99.5%), and isopropanol (Macron, 99.5%), were used as the precursors, 1 m KOH (Honeywell, > 99%) was used as the electrolyte, and Nafion solution (DuPont, 5%) was used as the binder without further purification.

##### Materials Synthesis

HEG was synthesized using a facile solvothermal method. In a typical process, equimolar Co(NO_3_)_2_·6H_2_O, Cr(NO_3_)_3_·9H_2_O Fe(NO_3_)_3_·9H_2_O, Mn(NO_3_)_2_·6H_2_O, and Ni(NO_3_)_2_·6H_2_O (0.5 mmol each) were dissolved in isopropanol (34 mL) under continuously stirring. After that, 6 mL of glycerol was added under stirring for another 30 min to obtain a homogenous darkbrown solution. The mixture was then transferred to a 100 mL Teflon‐lined stainless‐steel autoclave and heated at 150 °C for 10 h. After cooling down naturally, the powder product was collected by centrifuging, washing with absolute ethanol several times, and drying at 60 °C for 24 h in a vacuum oven. Sample thus obtained is designated as FeNiCoCrMn‐G. For comparison, the following glycerates were also prepared using the same method: Four‐metal glycerates, including ones without Cr (FeNiCoMn‐G), without Mn (FeNiCoCr‐G), without Fe (NiCoCrMn‐G), without Ni (FeCoCrMn‐G), and without Co (FeNiCrMn‐G). Three‐metal glycerate of Fe, Ni, and Co (FeNiCo‐G), two‐metal glycerate of Fe and Ni (FeNi‐G), and unary‐metal glycerates of Fe (Fe‐G), Ni (Ni‐G), and Co (Co‐G) were also prepared using the same method.

##### Material Characterizations

The crystalline structure was examined using X‐ray diffractometry (Bruker, D8 Discover) with a Cu K*α* radiation source having a wavelength of 1.5418 Å. The scanning angle was from 10° to 80° with a scanning rate of 2° min^−1^. The morphology was examined using SEM (JEOL 6701F) and TEM (JEOL JEM‐2100F). HRTEM equipped with SAED was used to investigate the microstructure. ICP‐MS (Thermo‐Element XR) and STEM‐EDS were used to analyze chemical composition. The surface chemistry was investigated using XPS (Versaprobe PHI 5000). The molecular structures of the obtained samples were examined using FTIR (PerkinElmer Frontier)

##### Electrode Preparation

The working electrode was prepared as follows: 5 mg of the catalyst powders and 40 µL of a 5 wt% Nafion solution were dispersed in a water (350 µL) and alcohol (150 µL) mixture under ultrasonication for at least 1h to form a homogeneous ink. The catalyst ink was then drop‐casted onto a Ni foam substrate covering an area of 1 × 1 cm. The electrode thus obtained was then dried in a vacuum oven at 60 °C for 1 h. The active mass loading was about 2.5 mg cm^−2^. Prior to use, the Ni foam was cleaned using a 3 m HCl solution for 10 min, and then sonicated in DI water, acetone, and ethanol subsequently for several times to remove the surface oxide.

##### Electrochemical Characterization

The electrochemical measurements were performed at room temperature using an Autolab electrochemical workstation (Muti Autolab/M204) with a built‐in EIS analyzer. The measurement was conducted with a standard three‐electrode cell where 1 m KOH was used as the electrolyte. A platinum foil and Ag/AgCl (3 m KCl) were used as the counter and reference electrodes, respectively. Before the measurement, the Ag/AgCl electrode was calibrated. The potential (*E*_Ag/AgCl_) was calibrated to the RHE potential (*E*_RHE_) following the equation of *E*_RHE_ = *E*_Ag/AgCl_ + 0.1976 + 0.059*pH. The pH of electrolyte was measured using a PHM 201 pH meter. Polarization curves were recorded under the LSV mode with a sweep rate of 5 mV s^−1^. All the curves were corrected with 80% IR compensation. AC impedance measurement was performed in a frequency range from 10^−1^ to 10^−5^ Hz with an AC amplitude of 10 mV. CV measurement was conduct in a non‐Faradaic region from of 0.95 to 1.05 V versus RHE at different scan rates to evaluate the *C*_dl_. Three independent electrodes were prepared and tested for each sample synthesis condition to obtain the average values and error margins. Long‐term durability of the electrode was determined via chronopotentiometric measurement for 36 h. The overall water splitting performance was also evaluated using a two‐electrode cell.

##### First‐Principles Calculations

Calculations based on the density functional theory (DFT) were performed using the Vienna Ab‐Initio Ssimulation software package (VASP),^[^ ^71^, ^72^ ^]^ with the projector augmented wave (PAW)^[^ ^71^, ^73^ ^]^ pseudopotential, and the generalized gradient approximation (GGA)^[^ ^74^ ^]^ for the exchange–correlation energy in the form of Perdew–Becke–Ernzehof. A 520 eV plane wave cutoff and the 2 × 2 × 2, 2 × 2 × 2 and 2 × 2 × 1 gamma–centered Monkhorst–Pack k–point grid were used for the calculations of FeNiCoMn–G, NiCoCrMn–G, and FeNiCoCrMn–G. All atomic positions are relaxed until the sum of the absolute forces is less than 0.05 eV Å^−1^. Spin–polarization effects were considered.

## Conflict of Interest

The authors declare no conflict of interest.

## Supporting information

Supporting Information

## References

[1] N.‐T. Suen , S.‐F. Hung , Q. Quan , N. Zhang , Y.‐J. Xu , H. M. Chen , Chem. Soc. Rev. 2017, 46, 337. 28083578 10.1039/c6cs00328a

[2] E. Fabbri , M. Nachtegaal , T. Binninger , X. Cheng , B.‐J. Kim , J. Durst , F. Bozza , T. Graule , R. Schäublin , L. Wiles , Nat. Mater. 2017, 16, 925. 28714982 10.1038/nmat4938

[3] G. Fu , Y. Wang , Y. Tang , K. Zhou , J. B. Goodenough , J.‐M. Lee , ACS Mater. Lett. 2019, 1, 123.

[4] J. Liu , Y. Zheng , Y. Jiao , Z. Wang , Z. Lu , A. Vasileff , S. Z. Qiao , Small 2018, 14, 1704073. 10.1002/smll.201704073 29542284

[5] Y. Lee , J. Suntivich , K. J. May , E. E. Perry , Y. Shao‐Horn , J. Phys. Chem. Lett. 2012, 3, 399. 26285858 10.1021/jz2016507

[6] B. Zhang , X. Zheng , O. Voznyy , R. Comin , M. Bajdich , M. García‐Melchor , L. Han , J. Xu , M. Liu , L. Zheng , Science 2016, 352, 333. 27013427 10.1126/science.aaf1525

[7] W. T. Hong , M. Risch , K. A. Stoerzinger , A. Grimaud , J. Suntivich , Y. Shao‐Horn , Energy Environ. Sci. 2015, 8, 1404.

[8] O. Diaz‐Morales , I. Ledezma‐Yanez , M. T. Koper , F. Calle‐Vallejo , ACS Catal. 2015, 5, 5380.

[9] D. Friebel , M. W. Louie , M. Bajdich , K. E. Sanwald , Y. Cai , A. M. Wise , M.‐J. Cheng , D. Sokaras , T.‐C. Weng , R. Alonso‐Mori , J. Am. Chem. Soc. 2015, 137, 1305. 25562406 10.1021/ja511559d

[10] J. Zhang , J. Liu , L. Xi , Y. Yu , N. Chen , S. Sun , W. Wang , K. M. Lange , B. Zhang , J. Am. Chem. Soc. 2018, 140, 3876. 29518310 10.1021/jacs.8b00752

[11] J. B. Gerken , S. E. Shaner , R. C. Massé , N. J. Porubsky , S. S. Stahl , Energy Environ. Sci. 2014, 7, 2376.

[12] Y. Yang , L. Dang , M. J. Shearer , H. Sheng , W. Li , J. Chen , P. Xiao , Y. Zhang , R. J. Hamers , S. Jin , Adv. Energy Mater. 2018, 8, 1703189.

[13] A.‐L. Wang , H. Xu , G.‐R. Li , ACS Energy Lett. 2016, 1, 445.

[14] Y. Pi , Q. Shao , P. Wang , F. Lv , S. Guo , J. Guo , X. Huang , Angew. Chem., Int. Ed. 2017, 56, 4502. 10.1002/anie.201701533 28322493

[15] J. W. Yeh , S. K. Chen , S. J. Lin , J. Y. Gan , T. S. Chin , T. T. Shun , C. H. Tsau , S. Y. Chang , Adv. Eng. Mater. 2004, 6, 299.

[16] B. Cantor , I. Chang , P. Knight , A. Vincent , Mater. Sci. Eng. A 2004, 375, 213.

[17] M. W. Glasscott , A. D. Pendergast , S. Goines , A. R. Bishop , A. T. Hoang , C. Renault , J. E. Dick , Nat. Commun. 2019, 10, 2650 31201304 10.1038/s41467-019-10303-z PMC6570760

[18] W. Wang , JOM 2014, 66, 2067.

[19] C. M. Rost , E. Sachet , T. Borman , A. Moballegh , E. C. Dickey , D. Hou , J. L. Jones , S. Curtarolo , J.‐P. Maria , Nat. Commun. 2015, 6, 8485. 26415623 10.1038/ncomms9485 PMC4598836

[20] S. Jiang , T. Hu , J. Gild , N. Zhou , J. Nie , M. Qin , T. Harrington , K. Vecchio , J. Luo , Scr. Mater. 2018, 142, 116.

[21] A. Sarkar , Q. Wang , A. Schiele , M. R. Chellali , S. S. Bhattacharya , D. Wang , T. Brezesinski , H. Hahn , L. Velasco , B. Breitung , Adv. Mater. 2019, 31, 1806236. 10.1002/adma.201806236 30838717

[22] J. Zhou , J. Zhang , F. Zhang , B. Niu , L. Lei , W. Wang , Ceram. Interfaces 2018, 44, 22014.

[23] P. Sarker , T. Harrington , C. Toher , C. Oses , M. Samiee , J.‐P. Maria , D. W. Brenner , K. S. Vecchio , S. Curtarolo , Nat. Commun. 2018, 9, 1. 30478375 10.1038/s41467-018-07160-7 PMC6255778

[24] P. H. Mayrhofer , A. Kirnbauer , P. Ertelthaler , C. M. Koller , Scr. Mater. 2018, 149, 93.

[25] J. Gild , Y. Zhang , T. Harrington , S. Jiang , T. Hu , M. C. Quinn , W. M. Mellor , N. Zhou , K. Vecchio , J. Luo , Sci. Rep. 2016, 6, 37946. 27897255 10.1038/srep37946 PMC5126569

[26] T. Jin , X. Sang , R. R. Unocic , R. T. Kinch , X. Liu , J. Hu , H. Liu , S. Dai , Adv. Mater. 2018, 30, 1707512. 10.1002/adma.201707512 29687496

[27] R.‐Z. Zhang , F. Gucci , H. Zhu , K. Chen , M. J. Reece , Inorg. Chem. 2018, 57, 13027. 30256098 10.1021/acs.inorgchem.8b02379

[28] J. Gild , J. Braun , K. Kaufmann , E. Marin , T. Harrington , P. Hopkins , K. Vecchio , J. Luo , J. Materiomics 2019, 5, 337.

[29] A. Sarkar , L. Velasco , D. Wang , Q. Wang , G. Talasila , L. de Biasi , C. Kübel , T. Brezesinski , S. S. Bhattacharya , H. Hahn , Nat. Commun. 2018, 9, 3400. 30143625 10.1038/s41467-018-05774-5 PMC6109100

[30] D. Bérardan , S. Franger , A. Meena , N. Dragoe , J. Mater. Chem. A 2016, 4, 9536.

[31] Y. Zhang , T. T. Zuo , Z. Tang , M. C. Gao , K. A. Dahmen , P. K. Liaw , Z. P. Lu , Prog. Mater. Sci. 2014, 61, 1.

[32] E. P. George , D. Raabe , R. O. Ritchie , Nat. Rev. Mater. 2019, 4, 515.

[33] A. Sarkar , C. Loho , L. Velasco , T. Thomas , S. S. Bhattacharya , H. Hahn , R. Djenadic , Dalton Trans. 2017, 46, 12167. 28869641 10.1039/c7dt02077e

[34] D. Bérardan , S. Franger , D. Dragoe , A. K. Meena , N. Dragoe , Phys. Status Solidi RRL 2016, 10, 328.

[35] J. Zhang , J. Yan , S. Calder , Q. Zheng , M. A. McGuire , D. L. Abernathy , Y. Ren , S. H. Lapidus , K. Page , H. Zheng , Chem. Mater. 2019, 31, 3705.

[36] T. X. Nguyen , J. Patra , J.‐K. Chang , J.‐M. Ting , J. Mater. Chem. A 2020, 8, 18963.

[37] C. Oses , C. Toher , S. Curtarolo , Nat. Rev. Mater. 2020, 1.

[38] Z. Jin , J. Lv , H. Jia , W. Liu , H. Li , Z. Chen , X. Lin , G. Xie , X. Liu , S. Sun , Small 2019, 15, 1904180. 10.1002/smll.201904180 31596058

[39] D. Wang , Z. Liu , S. Du , Y. Zhang , H. Li , Z. Xiao , W. Chen , R. Chen , Y. Wang , Y. Zou , J. Mater. Chem. A 2019, 7, 24211.

[40] T. Wang , H. Chen , Z. Yang , J. Liang , S. Dai , J. Am. Chem. Soc. 2020, 142, 4550. 32105461 10.1021/jacs.9b12377

[41] L. Shen , L. Yu , H. B. Wu , X.‐Y. Yu , X. Zhang , X. W. D. Lou , Nat. Commun. 2015, 6, 1. 10.1038/ncomms7694 25798849

[42] Y. Wang , L. Yu , X. W. Lou , Angew. Chem., Int. Ed. 2016, 55, 7423. 10.1002/anie.201601673 27095261

[43] F. X. Ma , H. Hu , H. B. Wu , C. Y. Xu , Z. Xu , L. Zhen , X. W. Lou , Adv. Mater. 2015, 27, 4097. 26038182 10.1002/adma.201501130

[44] Y. V. Kaneti , R. R. Salunkhe , N. L. W. Septiani , C. Young , X. Jiang , Y.‐B. He , Y.‐M. Kang , Y. Sugahara , Y. Yamauchi , J. Mater. Chem. A 2018, 6, 5971.

[45] D. Larcher , G. Sudant , R. Patrice , J.‐M. Tarascon , Chem. Mater. 2003, 15, 3543.

[46] M. Wang , J. Jiang , L. Ai , ACS Sustainable Chem. Eng. 2018, 6, 6117.

[47] Z. Dong , W. Zhang , Y. Xiao , Y. Wang , C. Luan , C. Qin , Y. Dong , M. Li , X. Dai , X. Zhang , ACS Sustainable Chem. Eng. 2020, 8, 5464.

[48] C. Zhang , B. Zhang , Z. Li , J. Hao , ACS Appl. Energy Mater. 2019, 2, 3343.

[49] X. Long , Z. Wang , S. Xiao , Y. An , S. Yang , Mater. Today 2016, 19, 213.

[50] E. Detsi , J. B. Cook , B. K. Lesel , C. L. Turner , Y.‐L. Liang , S. Robbennolt , S. H. Tolbert , Energy Environ. Sci. 2016, 9, 540. 30976318 10.1039/C5EE02509E PMC6456064

[51] D. Y. Chung , P. P. Lopes , P. F. B. D. Martins , H. He , T. Kawaguchi , P. Zapol , H. You , D. Tripkovic , D. Strmcnik , Y. Zhu , Nat. Energy 2020, 5, 222.

[52] L. Shen , L. Yu , X. Y. Yu , X. Zhang , X. W. Lou , Angew. Chem., Int. Ed. 2015, 54, 1868. 10.1002/anie.201409776 25522266

[53] N. L. W. Septiani , Y. Kaneti , K. B. Fathoni , G. Yanna , Y. Ide , B. Yuliarto , X. Jiang , N. Nugraha , H. K. Dipojono , D. Golberg , J. Mater. Chem. A 2020, 8, 30353047.

[54] Q. Wang , L. Shang , R. Shi , X. Zhang , Y. Zhao , G. I. Waterhouse , L. Z. Wu , C. H. Tung , T. Zhang , Adv. Energy Mater. 2017, 7, 1700467.

[55] X. Zheng , Y. Zhang , H. Liu , D. Fu , J. Chen , J. Wang , C. Zhong , Y. Deng , X. Han , W. Hu , Small 2018, 14, 1803666. 10.1002/smll.201803666 30307691

[56] A. Ramírez , P. Hillebrand , D. Stellmach , M. M. May , P. Bogdanoff , S. Fiechter , J. Phys. Chem. C 2014, 118, 14073.

[57] C. S. Lim , C. K. Chua , Z. Sofer , K. Klímová , C. Boothroyd , M. Pumera , J. Mater. Chem. A 2015, 3, 11920.

[58] K. Fan , H. Zou , Y. Lu , H. Chen , F. Li , J. Liu , L. Sun , L. Tong , M. F. Toney , M. Sui , ACS Nano 2018, 12, 12369. 30508382 10.1021/acsnano.8b06312

[59] N. C. Martins , J. Ângelo , A. V. Girão , T. Trindade , L. Andrade , A. Mendes , Appl. Catal., B 2016, 193, 67.

[60] M. S. Burke , M. G. Kast , L. Trotochaud , A. M. Smith , S. W. Boettcher , J. Am. Chem. Soc. 2015, 137, 3638. 25700234 10.1021/jacs.5b00281

[61] L. Trotochaud , S. L. Young , J. K. Ranney , S. W. Boettcher , J. Am. Chem. Soc. 2014, 136, 6744. 24779732 10.1021/ja502379c

[62] B. J. Trześniewski , O. Diaz‐Morales , D. A. Vermaas , A. Longo , W. Bras , M. T. Koper , W. A. Smith , J. Am. Chem. Soc. 2015, 137, 15112. 26544169 10.1021/jacs.5b06814

[63] H.‐J. Qiu , G. Fang , J. Gao , Y. Wen , J. Lv , H. Li , G. Xie , X. Liu , S. Sun , ACS Mater. Lett. 2019, 1, 526.

[64] J. Ryu , N. Jung , J. H. Jang , H.‐J. Kim , S. J. Yoo , ACS Catal. 2015, 5, 4066.

[65] H. Han , H. Choi , S. Mhin , Y.‐R. Hong , K. M. Kim , J. Kwon , G. Ali , K. Y. Chung , M. Je , H. N. Umh , Energy Environ. Sci. 2019, 12, 2443.

[66] G. Chen , Y. Zhu , H. M. Chen , Z. Hu , S. F. Hung , N. Ma , J. Dai , H. J. Lin , C. T. Chen , W. Zhou , Adv. Mater. 2019, 31, 1900883. 10.1002/adma.201900883 31099042

[67] Y. Liu , Q. Li , R. Si , G. D. Li , W. Li , D. P. Liu , D. Wang , L. Sun , Y. Zhang , X. Zou , Adv. Mater. 2017, 29, 1606200. 10.1002/adma.201606200 28128868

[68] H. Zhou , F. Yu , Q. Zhu , J. Sun , F. Qin , L. Yu , J. Bao , Y. Yu , S. Chen , Z. Ren , Energy Environ. Sci. 2018, 11, 2858.

[69] Z. W. Gao , T. Ma , X. M. Chen , H. Liu , L. Cui , S. Z. Qiao , J. Yang , X. W. Du , Small 2018, 14, 1800195. 10.1002/smll.201800195 29577621

[70] H. Zhang , B. Chen , H. Jiang , X. Duan , Y. Zhu , C. Li , Nanoscale 2018, 10, 12991. 29971287 10.1039/c8nr04195d

[71] G. Kresse , J. Furthmüller , Comput. Mater. Sci. 1996, 6, 15.

[72] G. Kresse , J. Furthmüller , Phys. Rev. B 1996, 54, 11169. 10.1103/physrevb.54.11169 9984901

[73] P. E. Blöchl , Phys. Rev. B 1994, 50, 17953. 10.1103/physrevb.50.17953 9976227

[74] J. P. Perdew , K. Burke , M. Ernzerhof , Phys. Rev. Lett. 1996, 77, 3865. 10062328 10.1103/PhysRevLett.77.3865

