# Supplementary material for: A New High Entropy Glycerate for High Performance Oxygen Evolution Reaction
Source: Adv Sci (Weinh). 2021 Jan 27;8(6):2002446. doi: 10.1002/advs.202002446 (PMC7967045; doi:10.1002/advs.202002446)
Supplement: Supplementary file 1 — Supporting Information [file ADVS-8-2002446-s001.pdf]

© 2020 Wiley-VCH GmbH

ADVANCED SCIENCE
Open Access

Supporting Information

for Adv. Sci., DOI: 10.1002/advs.202002446

A New High Entropy Glycerate for High Performance Oxygen Evolution Reaction

Nguyen Thi Xuyen, Yen-Hsun Su,Chia-Chun Lin, Jrjeng Ruan, and Jyh-Ming Ting[*]

Supporting Information

A New High Entropy Glycerate for High Performance Oxygen Evolution Reaction

*Nguyen Thi Xuyen, Yen-Hsun Su,Chia-Chun Lin, Jrjeng Ruan, Jyh-Ming Ting**

Department of Materials Science and Engineering, National Cheng Kung University
1 University Road, Tainan 70101, Taiwan

Email: jting@mail.ncku.edu.tw

Table S1. ICP analysis of the obtained samples.

Sample ID	Fe (at.%)	Ni (at.%)	Co (at.%)	Cr (at.%)	Mn (at.%)
FeNiG	51.3	48.7	0	0	0
FeNiCo-G	34.5	32.9	32.6	0	0
FeNiCoMn-G	27.1	25.6	26.0	0	21.3
FeNiCoCr-G	25.7	23.9	23.5	26.9	0
FeNiCrMn-G	26.9	0	24.4	28.2	20.5
FeCoCrMn-G	26.8	24	0	28.6	20.6
NiCoCrMn-G	0	26.1	25.2	28.4	20.3
FeNiCoCrMn-G	22.0	19.4	19.1	23.0	16.5

Table S2. Comparison of OER performances.

Electrocatalyst	Electrolyte	J (mA cm^{-2})	η_{10} (mV) @j	Tafel (mV/dec)	Ref
FeNiCoCrMn-G	1M KOH	10	229	40	This work
CoFeLaNiPt HEMG-NP	0.1 M KOH	10	377	-	[1]
$(CoCuFeMnNi)_3O_4$/MWCNT	1M KOH	10	350	59.5	[2]
AlNiCoFeMo- HEA	1M KOH	10	246	46	[3]
FeNi-Gly	1M KOH	10	320	50	[4]
CoFe-LDH/MXene	1M KOH	10	319	50	[5]
CTGU-10 MOF	1M KOH	10	240	58	[6]
EG/$Co_{0.85}$Se/NiFe-LDH	1M KOH	10	265	160	[7]
FeNi-HDNAs	1M KOH	10	206	91.66	[8]

NiCoFe-LDH	0.1M KOH	10	340	93	[9]
CoFeMo (oxy)hydroxides	1M KOH	10	277	28	[10]
NiFeMn LDH	1M KOH	20	289	47	[11]
NiFe-LDH/NF	1M KOH	10	210	58.9	[12]
NiFeCr LDH@CP	1M KOH	25	225	69	[13]
Exfoliated NiFe LDH	1M KOH	10	300	40	[14]
FeCoNiP	1M KOH	10	268	66	[15]
HE-MOF-RT	1M KOH	10	245	54	[16]
$K_{0.8}Na_{0.2}(MgMnFeCoNi)F_3$	1M KOH	10	314	57	[17]
AlFeCoNiNb alloy/oxyhydroxide	1M KOH	10	240	50	[18]

Table S3. Calculated strains in NiCoCrMn-G, FeNiCoMn-G, and FeNiCoCrMn-G

Average strain (Å /atom)	FeNiCoMn-G	NiCoCrMn-G	FeNiCoCrMn-G
Layer 1	0.550	0.567	0.505
Layer 2	0.511	0.461	0.528
Layer 3	0.499	0.540	0.540
Layer 4	0.504	0.574	0.501
Layer 5	N/A	N/A	0.537
Average	0.516	0.536	0.522

Table S4. HER performances of the glycerate samples. The standard deviations were obtained based on 3 independent measurements.

Sample ID	η_{10} ($mA.cm^{-2}$)	Tafel slope (mV/dec)
Ni foam	255 ± 4	132 ± 4
FeNi-G	224 ± 5	192 ± 6
FeNiCo-G	254 ± 5	137 ± 5
FeNiCoMn-G	251 ± 6	130 ± 4

FeNiCoCr-G	273 ± 3	116 ± 5
FeNiCrMn-G	241 ± 2	119 ± 3
FeCoCrMn-G	259 ± 3	122 ± 3
NiCoCrMn-G	268 ± 8	113 ± 4
FeNiCoCrMn-G	210 ± 4	105 ± 3

Table S5. Overall water splitting performance survey.

Electrocatalyst	J (mA cm^{-2})	Voltage (V)@j	Stability (h)	Ref
FeNiCoCoCr-G	10	1.63	24	This work
Ni-Co-P	10	1.62	20	[19]
$NiCo_2O_4$	10	1.65	32	[20]
Co_1Mn_1CH/NF	10	1.67	14	[21]
Se-(NiCo)S/OH	10	1.6	80	[22]
NiFe LDH	10	1.7	N/A	[12]
EG/$Co_{0.85}$Se/NiFe – LDH	10	1.67	10	[23]
CuO@Ni/NiFe hydroxide	40	1.73	10	[24]
$Co(OH)_2$@CoSe	100	1.94	50	[25]
Co_xP	10	1.59	N/A	[26]
Fe/Zn-CoP	10	1.59	12	[27]

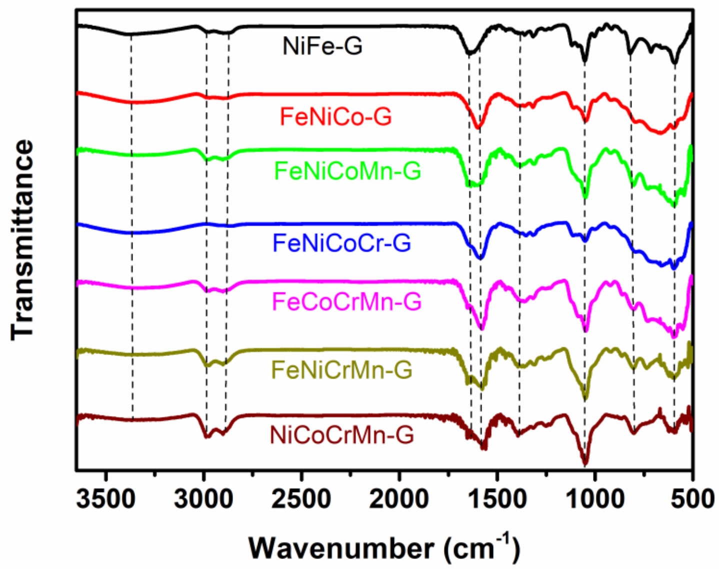

Figure S1. FTIR spectra of binary-, quaternary-, and quinary-metal glycerates.

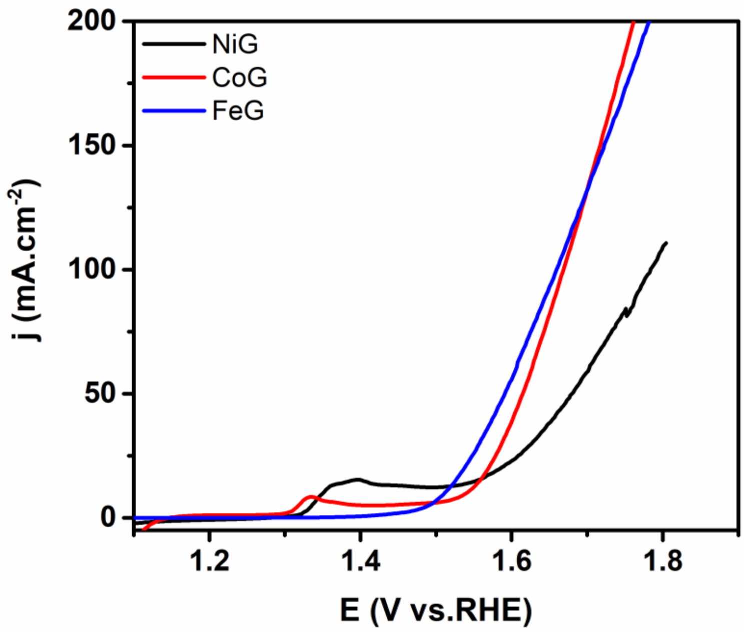

Figure S2. LSV curves of unary-metal glycerates of Fe (Fe-G), Ni (Ni-G), and Co (Co-G).

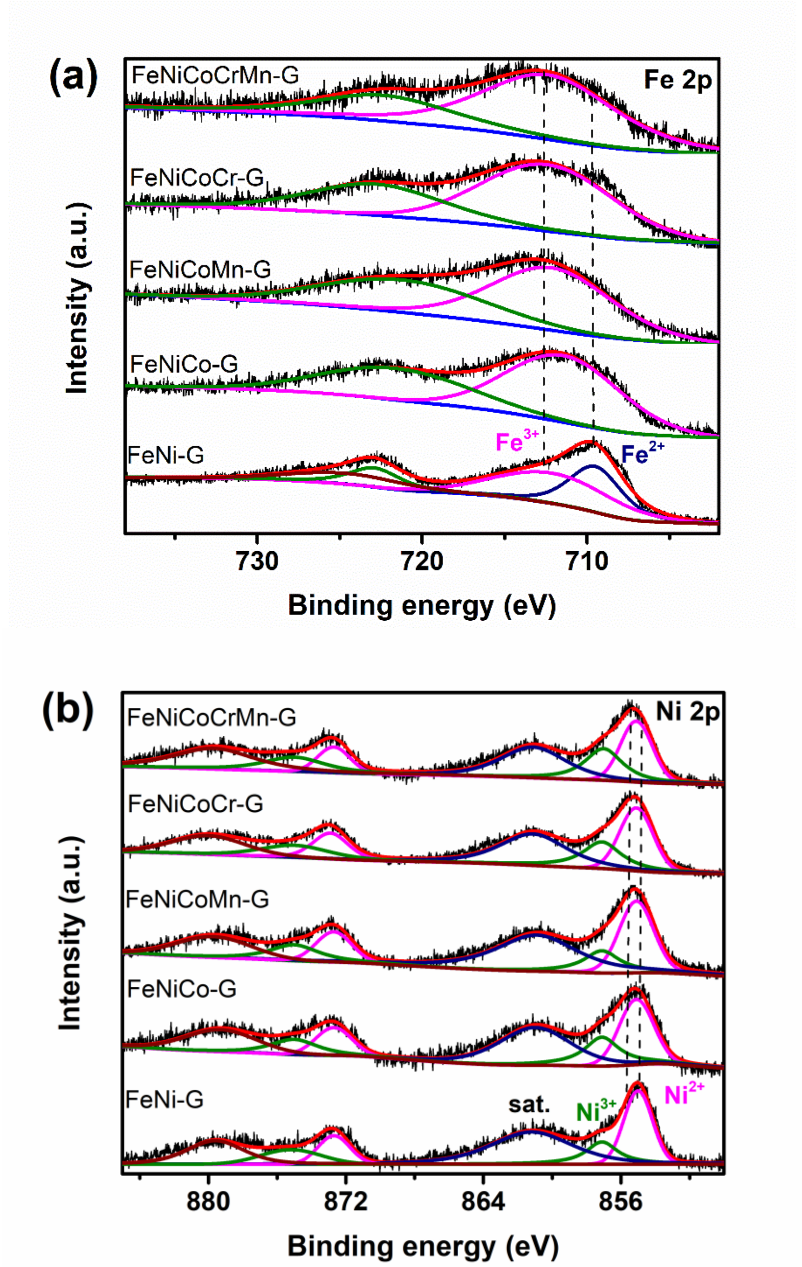

Figure S3. High resolution XPS (a) Fe 2p and (b) Ni 2p spectra of different glycerates.

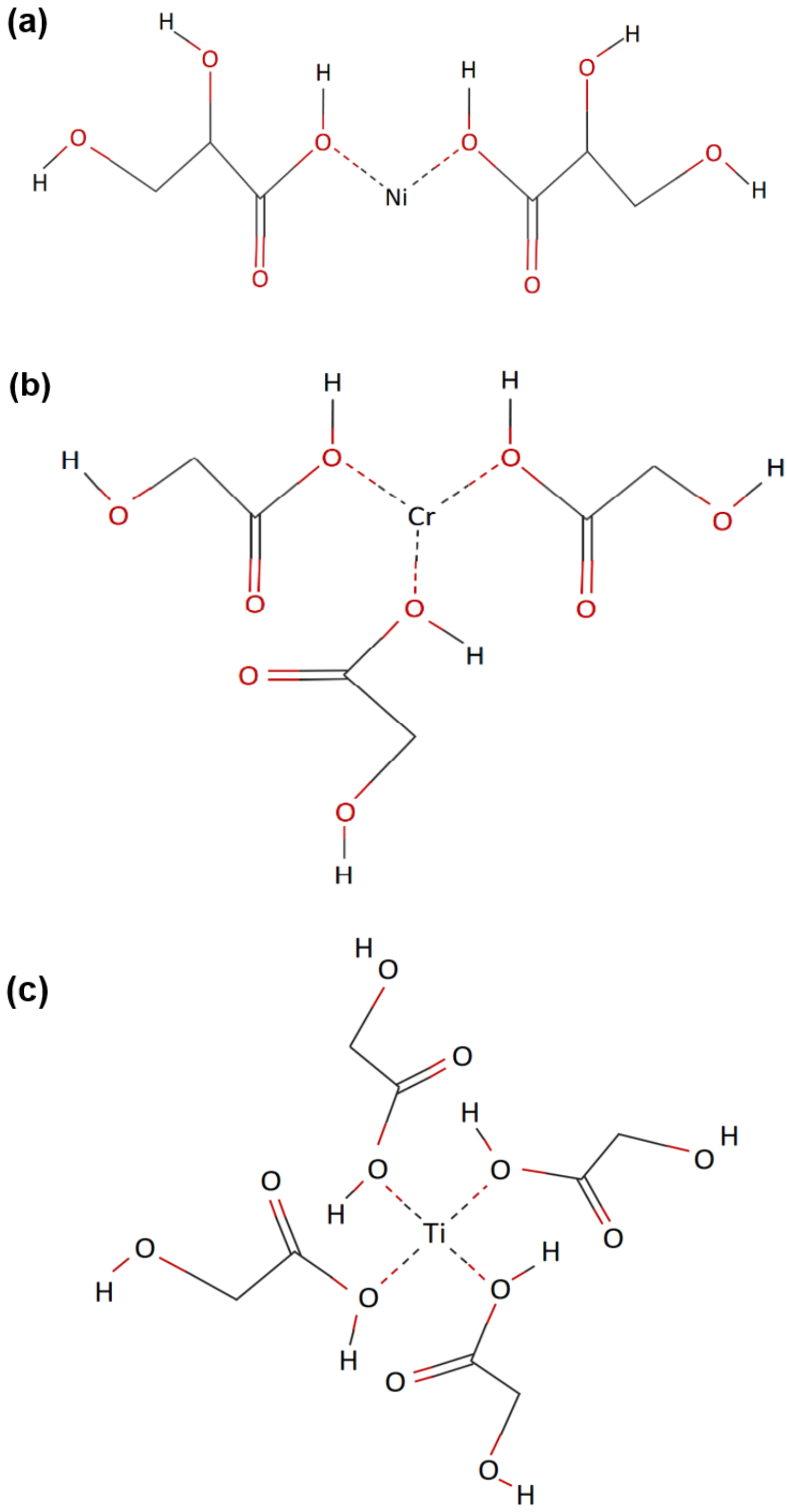

Figure S4. (a) Nickel-, (b) chromium-, and (c) titanium-based glycerates.

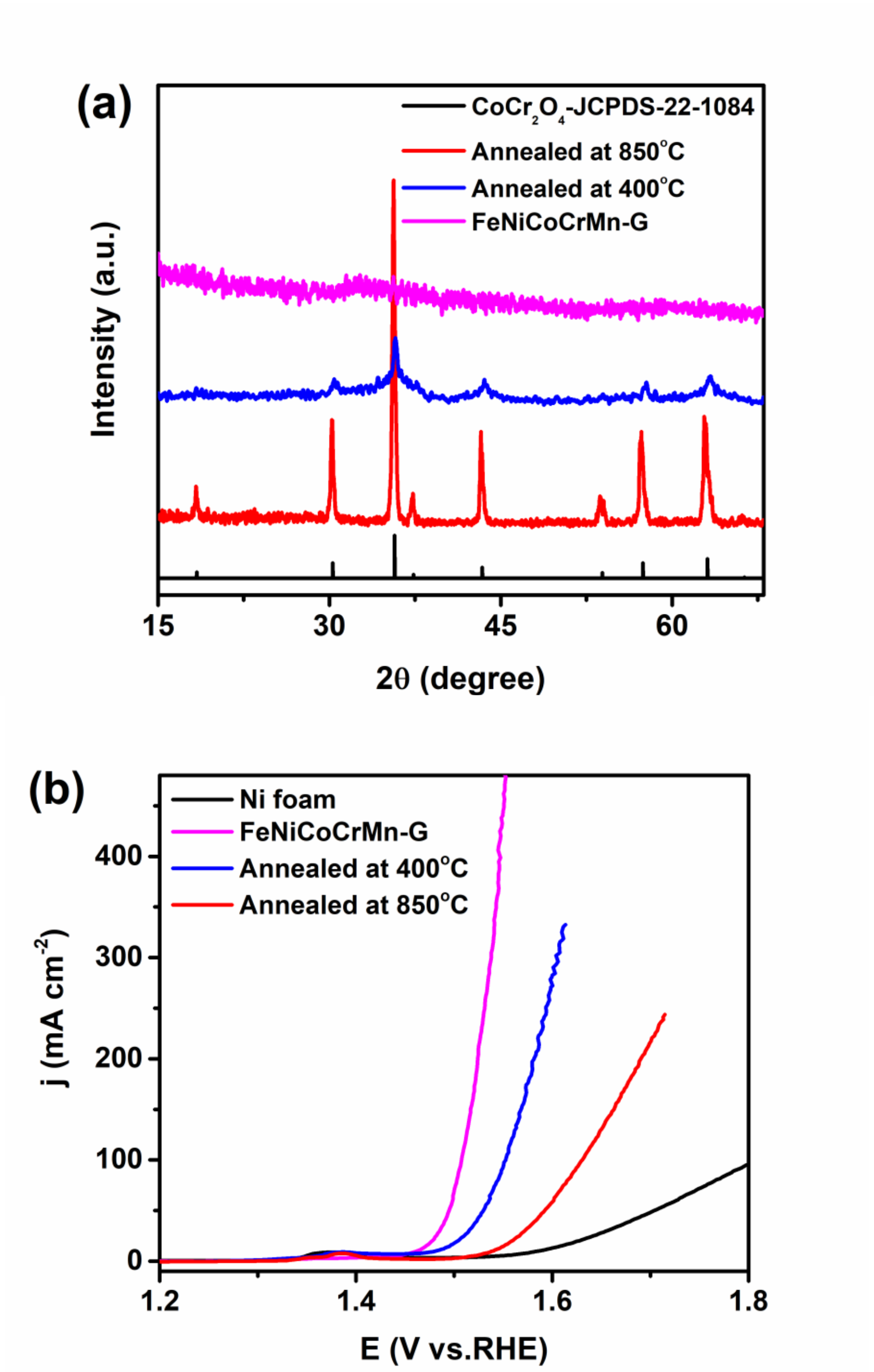

Figure S5. (a) XRD patterns, (b) OER polarization curves of as-synthesized and annealed FeNiCoCrMn-G samples. As-synthesized FeNiCoCrMn-G shows an amorphous structure, while the FeNiCoCrMn-Gs annealed at 400 and 850 ^{o}C exhibit a single-phase spinel structure.

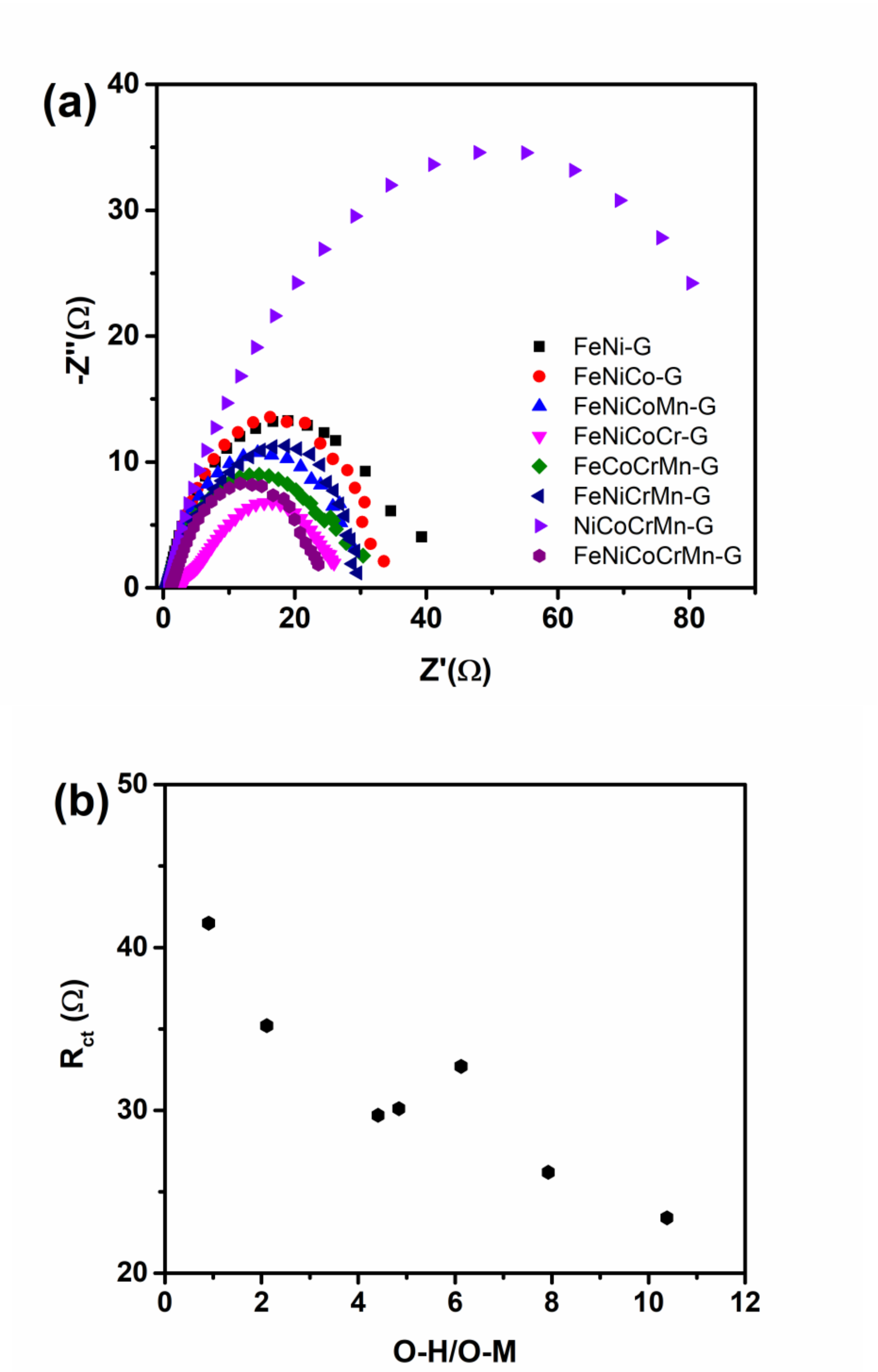

Figure S6. (a) Electrochemical impedance spectra measured at an overpotential of 320 mV. (b) R_{ct} decreases with the O-H/O-M ratio.

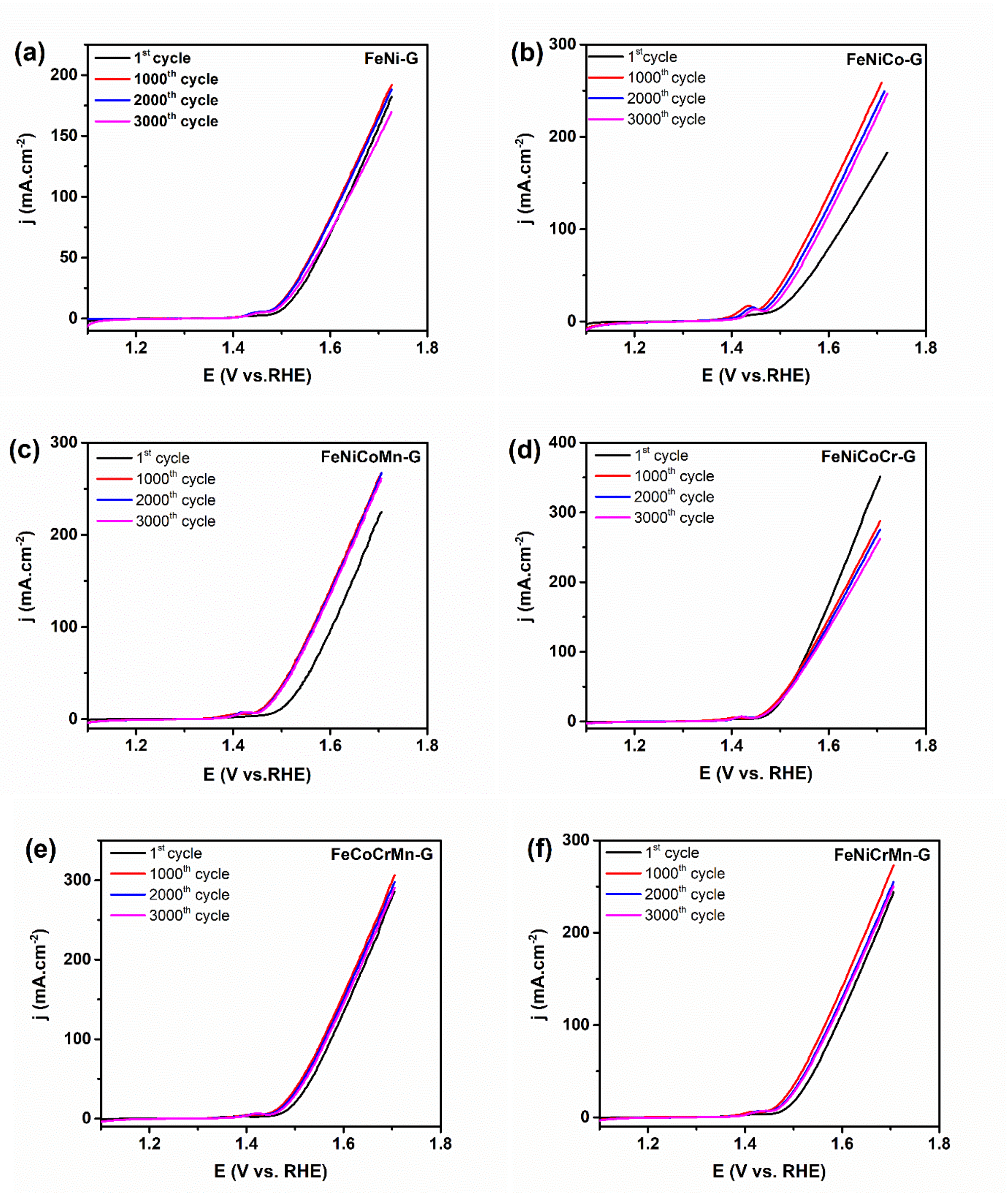
(a)
FeNi-G
1st cycle
1000th cycle
2000th cycle
3000th cycle
j (mA.cm-2)
E (V vs.RHE)
(b)
FeNiCo-G
1st cycle
1000th cycle
2000th cycle
3000th cycle
j (mA.cm-2)
E (V vs.RHE)
(c)
FeNiCoMn-G
1st cycle
1000th cycle
2000th cycle
3000th cycle
j (mA.cm-2)
E (V vs.RHE)
(d)
FeNiCoCr-G
1st cycle
1000th cycle
2000th cycle
3000th cycle
j (mA.cm-2)
E (V vs. RHE)
(e)
FeCoCrMn-G
1st cycle
1000th cycle
2000th cycle
3000th cycle
j (mA.cm-2)
E (V vs. RHE)
(f)
FeNiCrMn-G
1st cycle
1000th cycle
2000th cycle
3000th cycle
j (mA.cm-2)
E (V vs. RHE)

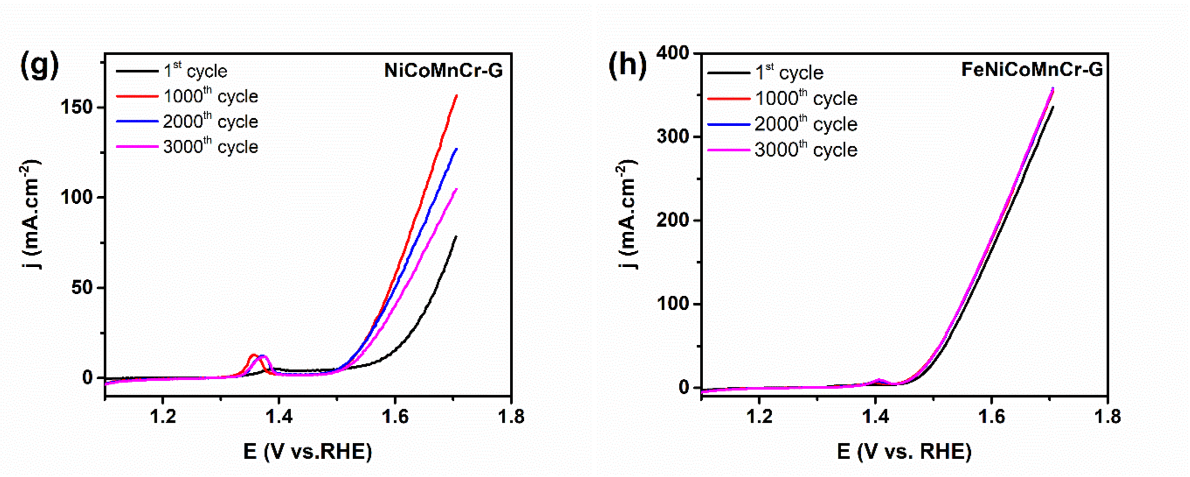

Figure S7. LSV curves after selected cycles of multi-metal gycerate electrocatalysts.

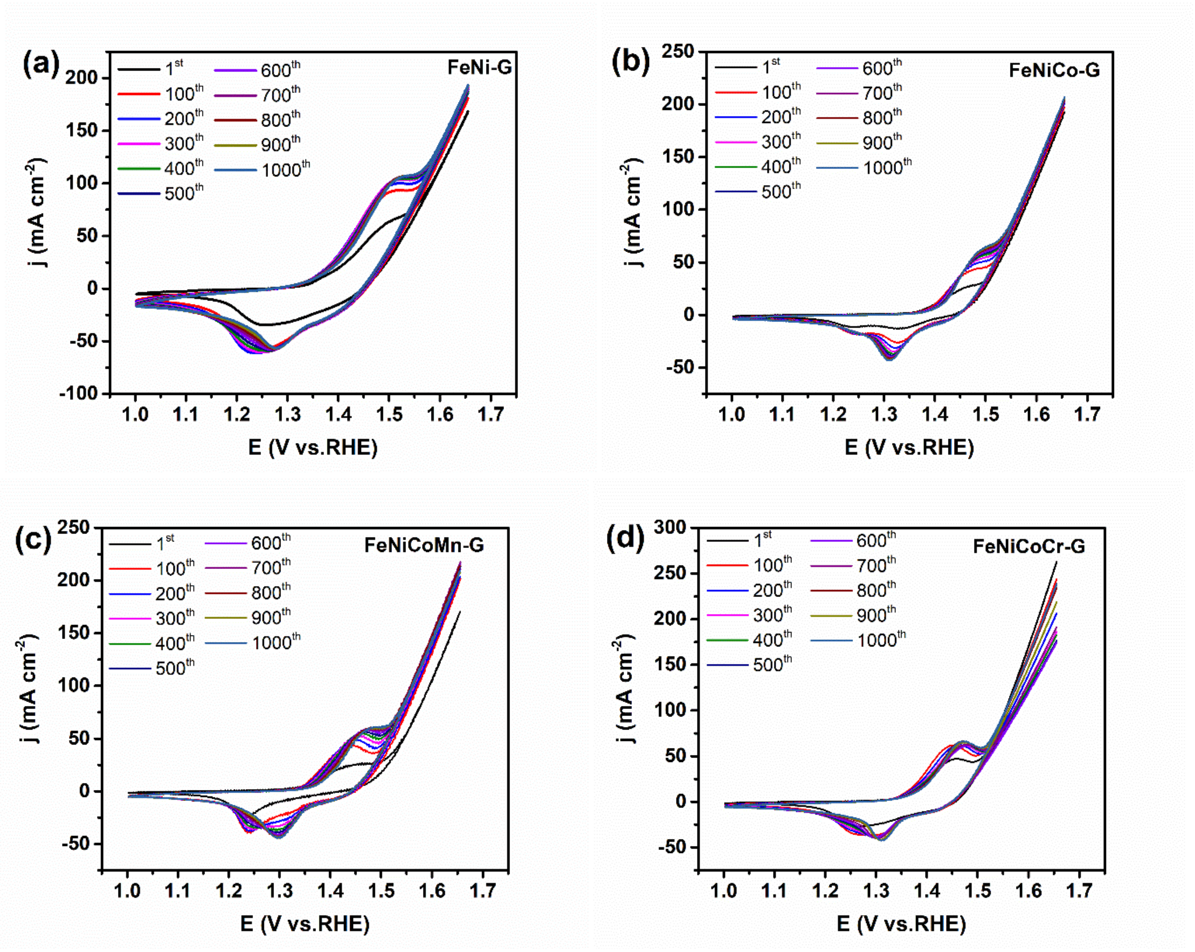

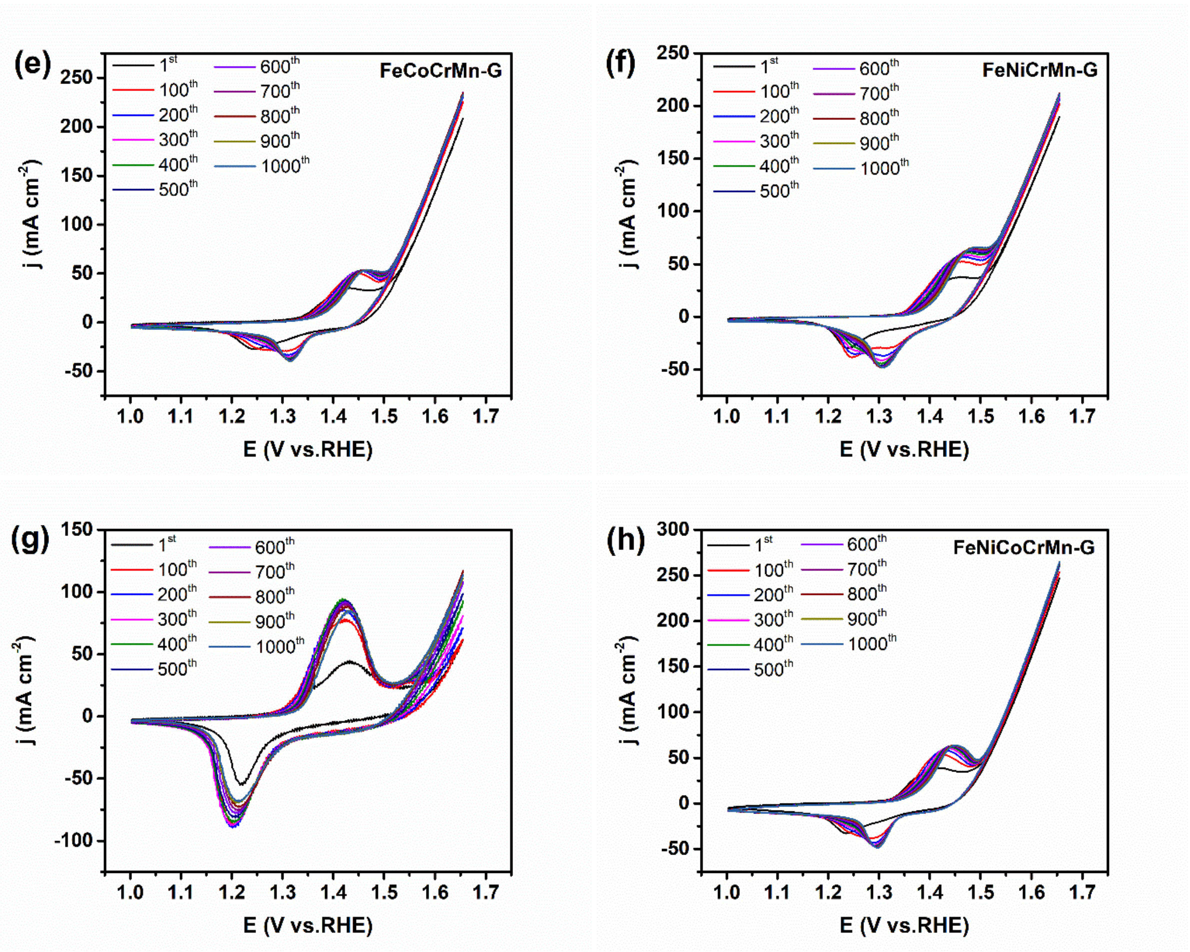

Figure S8. First 1000-cycle CV curves of glycerate electrodes.

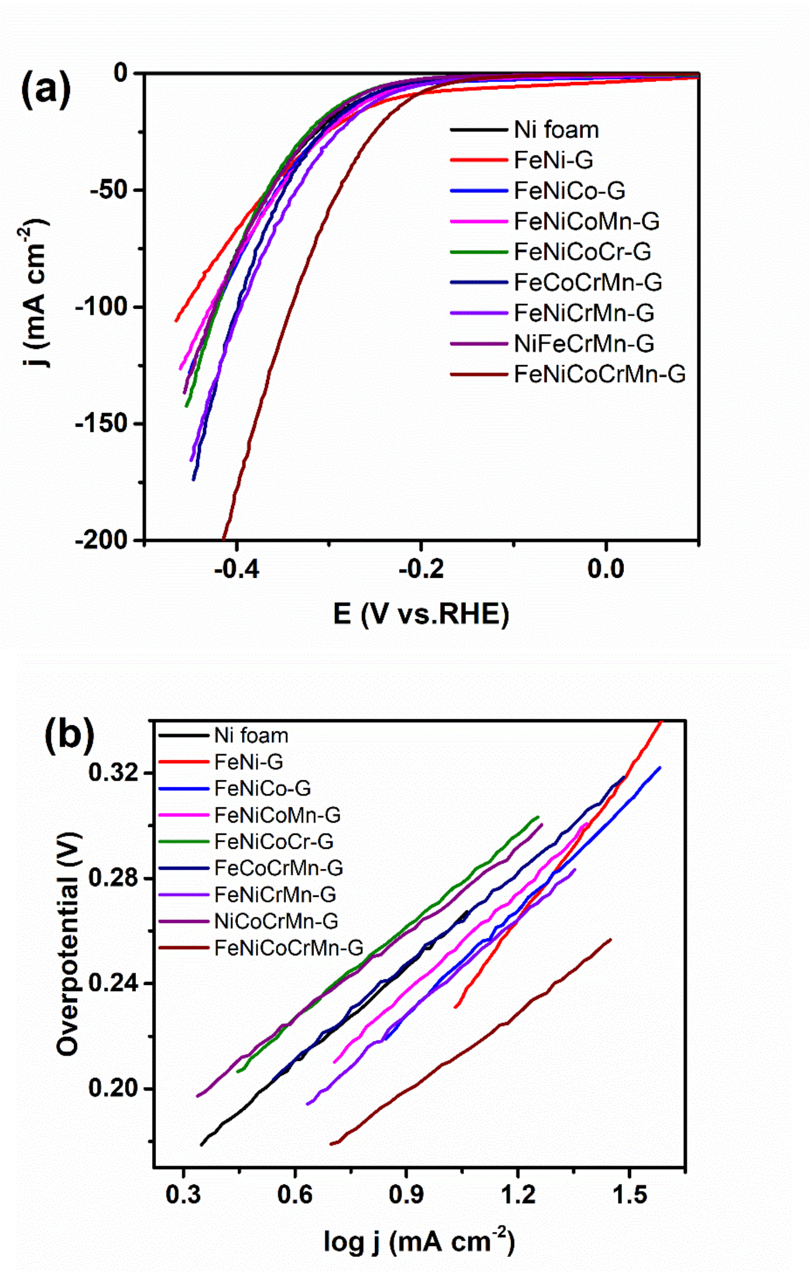

Figure S9. HER performances of the binary-, ternary-, quaternary-, and quinary-metal glycerates: (a) LSV curves and (b) Tafel plots.

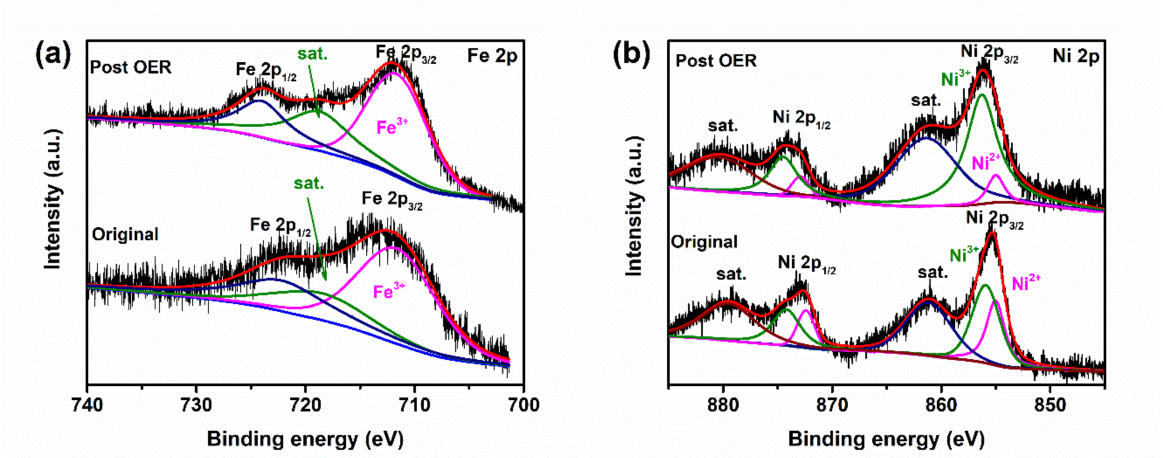

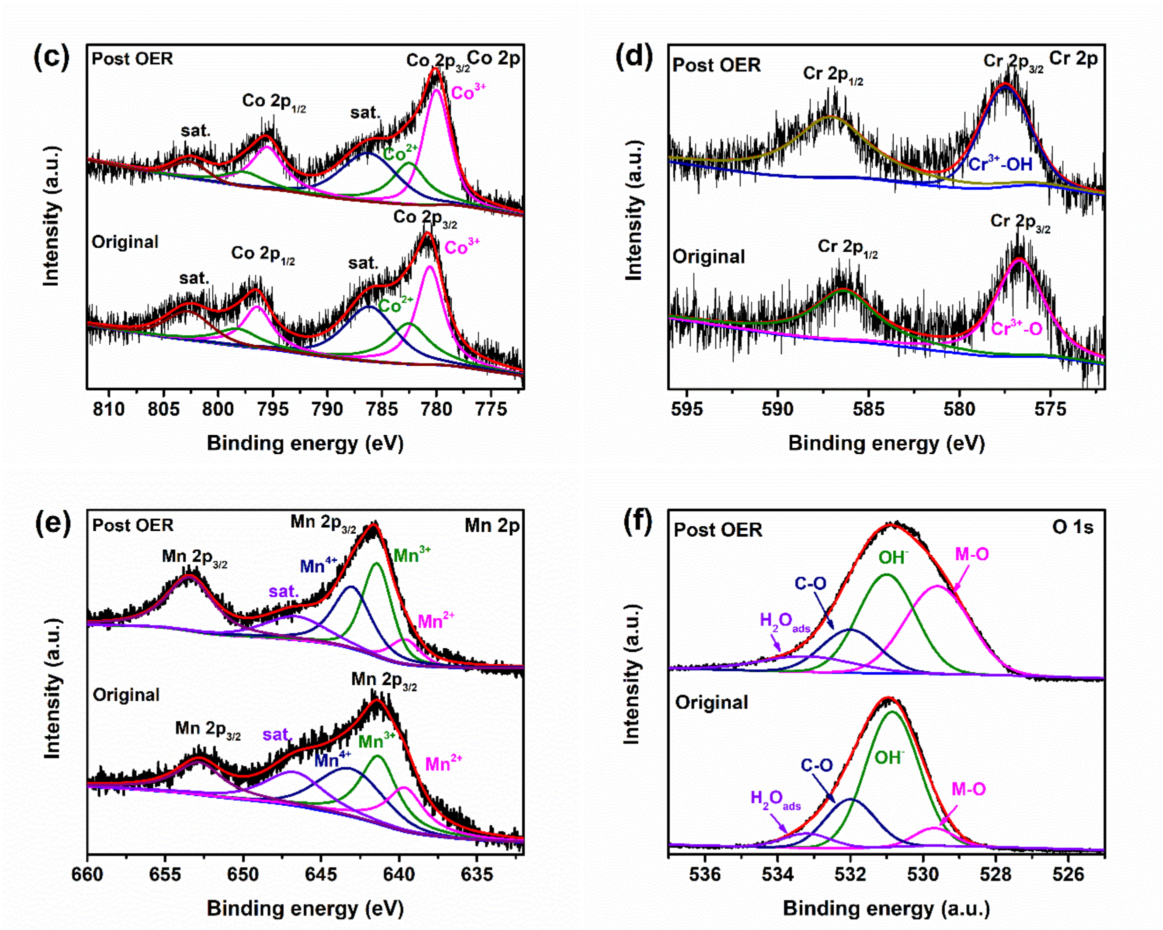

Figure S10. High resolution XPS spectra of the pristine and post-OER electrocatalysts. (a) Fe 2p, (b) Ni 2p, (c) Co 2p, (d) Cr 2p, (e) Mn 2p, and (f) O 1s spectra.

Reference

[1] M. W. Glasscott, A. D. Pendergast, S. Goines, A. R. Bishop, A. T. Hoang, C. Renault, J. E. Dick, *Nat. Commun.* **2019**, *10*, 1.

[2] D. Wang, Z. Liu, S. Du, Y. Zhang, H. Li, Z. Xiao, W. Chen, R. Chen, Y. Wang, Y. Zou, *J. Mater. Chem. A* **2019**, *7*, 24211.

[3] H.-J. Qiu, G. Fang, J. Gao, Y. Wen, J. Lv, H. Li, G. Xie, X. Liu, S. Sun, *ACS Mater. Lett.* **2019**, *1*, 526.

[4] M. Wang, J. Jiang, L. Ai, *ACS Sustain. Chem. Eng.* **2018**, *6*, 6117.

[5] C. Hao, Y. Wu, Y. An, B. Cui, J. Lin, X. Li, D. Wang, M. Jiang, Z. Cheng, S. Hu, *Mater. Today Energy* **2019**, *12*, 453.

[6] W. Zhou, D. D. Huang, Y. P. Wu, J. Zhao, T. Wu, J. Zhang, D. S. Li, C. Sun, P. Feng, X. Bu, *Angew. Chem. Int. Ed.* **2019**, *58*, 4227.

[7] Y. Tan, H. Wang, P. Liu, Y. Shen, C. Cheng, A. Hirata, T. Fujita, Z. Tang, M. Chen, *Energy Environ. Sci.* **2016**, *9*, 2257.

[8] N. Yu, W. Cao, M. Huttula, Y. Kayser, P. Hoenicke, B. Beckhoff, F. Lai, R. Dong, H. Sun, B. Geng, *Appl. Catal. B. Environ.* **2020**, *261*, 118193.

[9] L. Qian, Z. Lu, T. Xu, X. Wu, Y. Tian, Y. Li, Z. Huo, X. Sun, X. Duan, *Adv. Energy Mater.* **2015**, *5*, 1500245.

[10] P. F. Liu, S. Yang, L. R. Zheng, B. Zhang, H. G. Yang, *Chem. Sci.* **2017**, *8*, 3484.

[11] Z. Lu, L. Qian, Y. Tian, Y. Li, X. Sun, X. Duan, *ChemComm* **2016**, *52*, 908.

[12] J. Luo, J.-H. Im, M. T. Mayer, M. Schreier, M. K. Nazeeruddin, N.-G. Park, S. D. Tilley, H. J. Fan, M. Grätzel, *Sci* **2014**, *345*, 1593.
[13] Y. Yang, L. Dang, M. J. Shearer, H. Sheng, W. Li, J. Chen, P. Xiao, Y. Zhang, R. J. Hamers, S. Jin, *Adv. Energy Mater.* **2018**, *8*, 1703189.
[14] F. Song, X. Hu, *Nat. Commun.* **2014**, *5*, 1.
[15] J. Xu, J. Li, D. Xiong, B. Zhang, Y. Liu, K.-H. Wu, I. Amorim, W. Li, L. Liu, *Chem. Sci.* **2018**, *9*, 3470.
[16] X. Zhao, Z. Xue, W. Chen, X. Bai, R. Shi, T. Mu, *J. Mater. Chem. A* **2019**, *7*, 26238.
[17] T. Wang, H. Chen, Z. Yang, J. Liang, S. Dai, *J. Am. Chem. Soc.* **2020**, *142*, 4550.
[18] G. Fang, J. Gao, J. Lv, H. Jia, H. Li, W. Liu, G. Xie, Z. Chen, Y. Huang, Q. Yuan, *Appl. Catal. B. Environ.* **2020**, *268*, 118431.
[19] E. Hu, Y. Feng, J. Nai, D. Zhao, Y. Hu, X. W. D. Lou, *Energy Environ. Sci.* **2018**, *11*, 872.
[20] X. Gao, H. Zhang, Q. Li, X. Yu, Z. Hong, X. Zhang, C. Liang, Z. Lin, *Angew. Chem. Int. Ed.* **2016**, *55*, 6290.
[21] T. Tang, W.-J. Jiang, S. Niu, N. Liu, H. Luo, Y.-Y. Chen, S.-F. Jin, F. Gao, L.-J. Wan, J.-S. Hu, *J. Am. Chem. Soc.* **2017**, *139*, 8320.
[22] C. Hu, L. Zhang, Z. J. Zhao, A. Li, X. Chang, J. Gong, *Adv. Mater.* **2018**, *30*, 1705538.
[23] Y. Hou, M. R. Lohe, J. Zhang, S. Liu, X. Zhuang, X. Feng, *Energy Environ. Sci.* **2016**, *9*, 478.
[24] Y. Liu, Z. Jin, X. Tian, X. Li, Q. Zhao, D. Xiao, *Electrochim. Acta* **2019**, *318*, 695.
[25] Y. Wang, Y. Yang, X. Wang, H. Shao, P.-H. Li, T. Li, H. Liu, Q. Zheng, J. Hu, L. Duan, *Nanoscale Advances* **2020**.
[26] H. Xue, H. Zhang, S. Fricke, M. Lüther, Z. Yang, A. Meng, W. Bremser, Z. Li, *Sustain. Energy Fuels* **2020**.
[27] P. Wang, X. Liu, Y. Yan, J. Cao, J. Feng, J. Qi, *Catal. Sci. Technol.* **2020**.
